# Diagnostic Potential of Plasma IgA1 O-Glycans in Discriminating IgA Nephropathy From Other Glomerular Diseases and Healthy Participants

**DOI:** 10.3389/fmolb.2022.871615

**Published:** 2022-04-04

**Authors:** Shuyu Zhang, Haidan Sun, Zejian Zhang, Menglin Li, Zhengguang Guo, Wenling Ye, Guangyan Cai, Wei Sun, Mingxi Li

**Affiliations:** ^1^ Department of Nephrology, State Key Laboratory of Complex Severe and Rare Diseases, Peking Union Medical College Hospital, Chinese Academy of Medical Science and Peking Union Medical College, Beijing, China; ^2^ Core Facility of Instruments, School of Basic Medicine, Peking Union Medical College, Institute of Basic Medical Sciences, Chinese Academy of Medical Sciences, Beijing, China; ^3^ Medical Research Center, State Key Laboratory of Complex Severe and Rare Diseases, Peking Union Medical College Hospital, Chinese Academy of Medical Science and Peking Union Medical College, Beijing, China; ^4^ State Key Laboratory of Bioactive Substances and Functions of Natural Medicines, Institute of Materia Medica, Peking Union Medical College, Chinese Academy of Medical Sciences, Beijing, China; ^5^ Department of Nephrology, The First Medical Centre, Chinese PLA General Hospital, Medical School of Chinese PLA, Beijing, China

**Keywords:** iga nephropathy, O-glycosylation, IgA1 hinge region, glycoproteomics, biomarker

## Abstract

**Background:** Aberrant O-glycosylation of IgA1 plays an important role in IgA nephropathy pathogenesis. Previous proteomic studies analyzed O-glycans of the circulating IgA1 hinge region and found that the N-acetylgalactosamine (GalNAc) and galactose numbers in the hinge region of IgA1 of patients with IgA nephropathy were lower than those in healthy participants. However, the diagnostic performance of the O-glycosylation traits in the hinge region of plasma IgA1 for IgA nephropathy remains unelucidated. The present study aimed to determine the difference in plasma IgA1 hinge region O-glycoforms among IgA nephropathy, non-IgA nephropathy disease controls, and healthy participants, and to further evaluate the diagnostic performance of plasma IgA1 glycosylation traits.

**Methods:** Sixty-two patients with biopsy-proven primary IgA nephropathy, 30 age- and sex-matched non-IgA nephropathy disease controls (10 patients with membranous nephropathy, 10 with focal segmental glomerulosclerosis, and 10 with minimal change disease), and 30 healthy participants were prospectively recruited. Plasma galactose deficient-IgA1 levels were measured using a KM55 kit. Plasma IgA was extracted using IgA immunoaffinity beads. After de-N-glycosylation, reduction, alkylation, trypsin digestion, and O-glycopeptide enrichment via hydrophilic interaction liquid chromatography, liquid chromatography tandem mass spectrometry (LC-MS/MS) was applied to analyze the IgA1 O-glycosylation patterns and we derived the plasma IgA1 O-glycosylation traits.

**Results:** Plasma IgA1 O-glycosylation patterns were significantly changed in IgA nephropathy patients compared to those with non-IgA nephropathy disease controls and healthy participants. The GalNAc number was lowest in IgA nephropathy patients. In addition, a similar result was observed for the galactose number in the IgA1 hinge region. These values showed moderate potential for discriminating between IgA nephropathy and the controls. When these values were combined, the area under the curve increased compared to when they were considered individually. When further adding a clinical indicator, the area under the curve of the GalNAc-galactose-IgA panel exceed 0.9 in discriminating IgA nephropathy from the controls.

**Conclusion:** The amount of GalNAc and galactose in plasma IgA1 hinge region identified by glycoproteomics could be used as a diagnostic biomarker for IgA nephropathy. The panel containing GalNAc, galactose, and circulating IgA displayed excellent diagnostic performance and is promising for practical clinical applications.

## Introduction

IgA nephropathy (IgAN) is the most common form of primary glomerulonephritis worldwide, and 20–30% of patients with IgAN progress to end-stage renal disease within 10 years of disease onset (Floege and Amann, 2016). Renal biopsy is the gold standard for diagnosing IgAN. It is characterized by IgA deposition, especially IgA1, in the mesangial area, which is related to mesangial proliferation. A renal biopsy is an invasive procedure however, there are no validated diagnostic serum or urine biomarkers of IgAN. Therefore, there is an urgent need to identify non-invasive biomarkers for the diagnosis of IgAN.

Human IgA1 has a hinge region (HR) containing 21 amino acids with nine potential O-glycosylation sites, and the occupancy of these sites is variable (Ohyama et al., 2021). IgA1 HR usually has fewer than six serine or threonine residues modified by O-linked glycans (Tarelli et al., 2004; Woof and Russell, 2011). The formation of O-linked glycans begins with the addition of N-acetylgalactosamine (GalNAc) followed by galactose (Gal), and both GalNAc and Gal can be modified by sialic acid (SA) (Field et al., 1989). Evidence suggests that IgAN is a consequence of abnormal O-glycosylation in the HR of IgA1, resulting in increased circulation of galactose-deficient IgA1 (Gd‐IgA1) (Moldoveanu et al., 2007; Allen et al., 2008). The exposed GalNAc in Gd-IgA1 HR as a trigger factor could form large immune complexes with anti-glycan IgG antibodies (Suzuki et al., 2009; Knoppova et al., 2016). Aberrant glycosylation of IgA1 molecules and glycan-specific antibody levels are considered the most promising biomarkers for IgAN diagnosis. The measurement of Gd-IgA1 in blood is usually based on lectin binding activity by an ELISA-type approach, which recognizes terminal GalNac residues on O-glycans (Tachibana et al., 2006; Moore et al., 2007). There may be significant differences when different batches of lectin reagents are used. An anti-Gd-IgA1 monoclonal antibody (KM55) specifically recognizes circulating Gd-IgA1, and an ELISA using KM55 was highly consistent with the lectin detection method (Yasutake et al., 2015). However, these methods cannot provide specific information on IgA1 O-glycans. Given the heterogeneity of IgA1 HR O-glycoforms, it is necessary to explore IgAN biomarkers from a glycoproteomic perspective.

Previous studies (Odani et al., 2000; Hiki et al., 2001) applied mass spectrometry, used the theoretical molecular weight method to infer the O-glycoforms of IgA1 HR, and manually quantified Gd-IgA1 O-glycopeptides. However, there remain some deviations in the deduction of the IgA1 HR glycoforms based on theoretical molecular weight and quantification according to glycopeptide peak intensity. Moreover, no clinical study has analyzed the diagnostic performance of O-glycosylation modification in circulating IgA1 HR for IgAN diagnosis.

We hypothesized that circulating IgA1 HR in patients with IgAN could have different glycoforms and unique glycosylation characteristics (GalNAc and Gal number) compared to patients with non-IgA nephropathy disease controls and healthy participants. In this study, we applied IgA affinity beads (Dotz et al., 2021) to extract plasma IgA and analyzed the O-glycosylation of plasma IgA1 HR in a single-center IgAN cohort using LC-electron-transfer higher-energy collision dissociation (EThcD)-MS/MS and automatic glycopeptide quantitative software. We aimed to determine the difference in plasma IgA1 HR O-glycoforms among patients with IgAN, non-IgAN disease controls (DCs), and healthy controls (HCs) and to investigate the diagnostic potential of the O-glycosylation traits of IgA1 HRs for IgAN.

## Methods and Materials

### Study Populations

**FIGURE 1:**
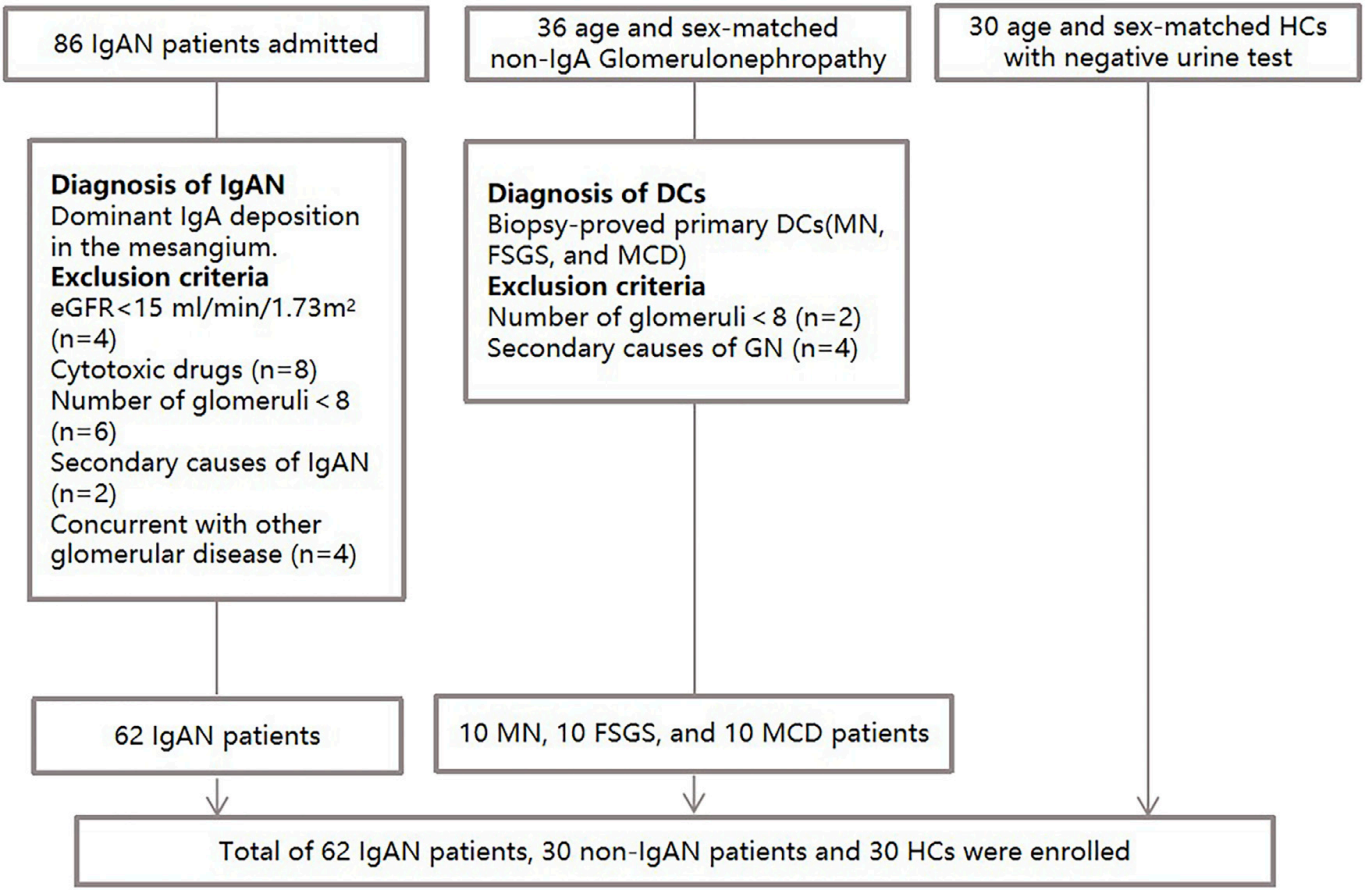
Flow diagram of the enrollment and exclusion criteria in the study population.

Plasma samples from 62 patients with biopsy-proven primary IgAN, 30 patients with non-IgAN DCs and 30 HCs were consecutively and prospectively collected between april 2020 and June 2021 from Peking Union Medical College Hospital (Beijing, China) (Figure 1). The age and gender of three groups of participants were matched as far as possible. The exclusion criteria were as follows: 1) Patients with secondary IgAN caused by IgA vasculitis, liver cirrhosis or lupus; 2) Patients received cytotoxic drug therapy; 3) The number of glomeruli in biopsy specimens was less than eight. HCs were defined by medical doctors according to the eligibility criteria and they had no history of hypertension, diabetes, no hematuria, proteinuria and kidney diseases. DCs included 10 membranous nephropathy patients, 10 with focal segmental glomerulosclerosis, and 10 with minimal change disease. The exclusion criteria of DC included 1) Patients with secondary glomerulonephropathy; 2) Patients with comorbid IgAN; 3) The number of glomeruli in biopsy specimens was less than eight. All work contained in this study was approved by the ethics committee of Peking Union Medical College Hospital (Number: JS-2573). All participants gave written informed consent in accordance with the ethical principles stated in the Declaration of Helsinki.

### Clinical and Pathological Data

Clinical manifestations at the time of renal biopsy were collected from medical records. The estimated glomerular filtration rate (eGFR) was calculated by the Chronic Kidney disease Epidemiology Collaboration equation (Levey and Stevens, 2010). The pathological diagnosis was based on the Oxford pathological classification (MESTC score) criteria, including mesangial hypercellularity (M0/M1), endocapillary hypercellularity (E0/E1), segmental glomerulosclerosis (S0/S1), tubular atrophy and interstitial fibrosis (T0/T1/T2), and crescent (C0/C1/C2) (Trimarchi et al., 2017).

### Plasma Gd-IgA1 Detection by Enzyme-Linked Immunosorbent Assay

Chemicals and instruments can be found in Supplementary Material S1. The Gd-IgA1 in plasma was measured by solid-phase sandwich enzyme immunoassay kit following the manufacturer’s instructions (Yasutake et al., 2015). Briefly, the wells were precoated with antibodies specific for Gd-IgA1 (KM55). Supernatant samples or standard of Gd-IgA1 were incubated in a plate for 2 h at 37°C, followed by washing and incubation with HRP-conjugated anti-human IgA1 antibody for 1 h at 37°C. Finally, the color was developed and measured at OD450/630 nm.

### IgA Enrichment

The IgA immunoaffinity beads were used for plasma IgA enrichment as previous study (Dotz et al., 2021). Fifty microliters of bead slurry was applied to EP tubes (1.5 ml, BioRad, CA, United States ) and washed three times with 200 μL PBS. For IgA capturing, we added 150 μL plasma into per EP tube, followed by 1 h incubation at room temperature with agitation. The beads were washed three times with 200 μL PBS and three times with 200 μL water, prior to centrifugation for 1 min at 1,000 g. For elution, 300 μL of 100 mM glycine was added, followed by incubation with agitation for 30 min. Eluates were collected into new EP tubes and dried by vacuum centrifugation at room temperature.

### De-N-glycosylation

For this procedure, 100 μg of IgA protein was resuspended in 45 μL deionized water and 5 μL protein denaturation buffer and heated at 100°C for 10 min. Then 10 μL of PNGase F was added, and the mixture was digested at 37°C overnight. The reaction was stopped by heating at 100°C for 2 min.

### Reduction, Alkylation, and Trypsin Digestion

The N-deglycosylated IgA was proteolyzed following the filter-aided sample preparation (FASP) protocol (Wiśniewski et al., 2009). The IgA was mixed with 200 μL uric acid buffer (8 M urea in 0.1 M Tris-HCl, pH 8.5). The solution was loaded onto an ultrafiltration cartridge and was reduced with 100 μL of 20 mM dithiothreitol for 4 h at 37°C, then alkylated with 100 μL of 50 mM iodoacetamide for 30 min at 25°C in the darkness. The buffer was subsequently replaced with 50 mM NH4HCO3 buffer by washing the ultrafiltration membrane thrice. The trypsin was added with an enzyme to substrate ratio of 1:50, and the solution was incubated overnight at 37°C. The digests were collected via centrifugation at 14,000 g for 15 min and stored at -80°C.

### Neuraminidase Treatment

The sialic acid was removed from IgA1 glycans using 10 mU/mL of neuraminidase from *Clostridium perfringens* in 50 mM sodium phosphate (pH 6.0) at 37°C overnight. Then the sample was collected by centrifugation and lyophilized until dry for glycopeptide enrichment.

### Intact O-Glycopeptide Enrichment *via* HILIC

Intact O-glycopeptides were enriched using a hydrophilic interaction liquid chromatography (HILIC) enrichment method (Zhang et al., 2018). Briefly, IgA tryptic peptide was resuspended briefly in 80% ACN/0.2% TFA. Then, 5 mg of HILIC column was washed three times for 10 min each with 0.1% TFA and 80% ACN/0.2% TFA, followed by sample loading and rotating for 2 h at room temperature. Then, the mixture was transferred to a homemade pipet tip that was packed with C8 membrane and washed twice with 80%ACN/0.2%TFA. IgA1 glycopeptides bound to the HILIC column were eluted three times with 70 μL of 0.1% TFA. The samples were dried by vacuum centrifugation.

### LC-MS/MS

**FIGURE 2:**
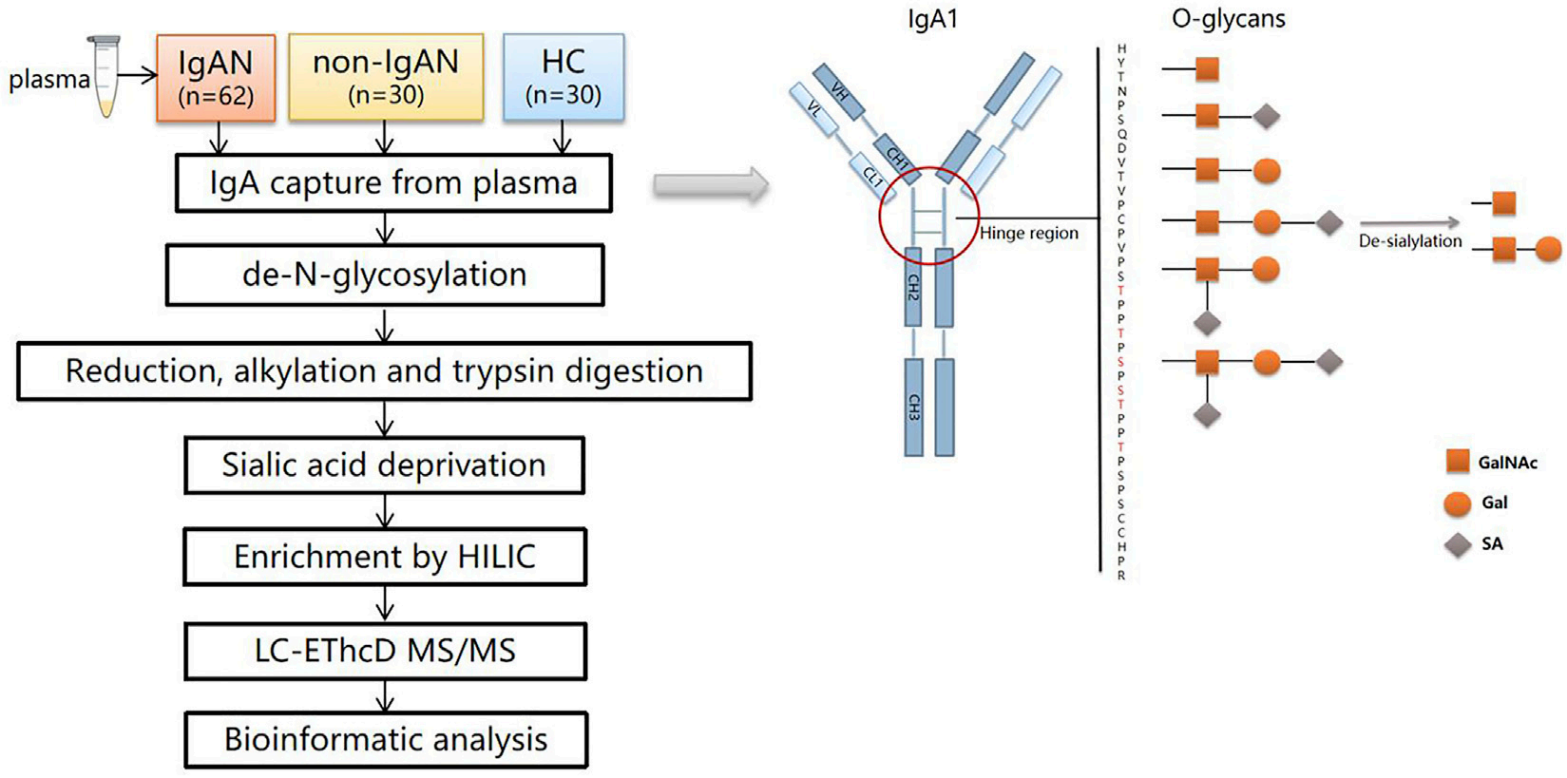
The workflow of the study and schematic view of IgA1 O-glycosylation. There are six types of O-glycans in the IgA1 HR consisting of N-acetylgalactosamine (GalNAc), galactose (Gal), and sialic acid (SA). After de-sialylation, two types of monosaccharides were left (☐:GalNAc, O: Gal).

The LC–MS/MS detection was carried out as reported previously (Zhang et al., 2018). The samples were dissolved in 0.1% FA and separated in a capillary column (150 μm × 120 mm) packed with C18 (1.9 μm) at a flow rate of 0.6 μL/min. Mobile phase A (2% ACN/0.1% FA) and mobile phase B (99.9% ACN/0.1% FA) were used, and the elution gradient was from 6 to 32% mobile phase B for 30 min. The glycopeptides were analyzed by an Orbitrap Fusion Lumos Tribrid Mass Spectrometer under EThcD mode. Data was acquired in data dependent acquisition mode. The MS1 was analyzed with a mass range of 400–1600 KDa at a resolution of 60,000. The automatic gain control (AGC) was set as 1e6 and the maximum injection time (MIT) was 150 m. The MS2 was analyzed in the data-dependent mode for the 20 most intense ions subjected to fragmentation in the Orbitrap. For each scan, the AGC was set at 5e4 and the MIT was set at 150 m. The supplement activation energy was 35%. The cycle time was 3s. The workflow of this study was summarized in Figure 2.

### Data Analysis

A UniProt human IgA1 database (P01876, https://www.uniprot.org/) was used for data searching in Byonic (Protein Metrics, San Carlos, CA) (Bern et al., 2012). The parameters used in database searching by Byonic were set as follows: carbamidomethylation was set as the fixed modification; N-terminal acylation, methionine oxidation, and deamidation of asparagine and glutamine were set as the variable modifications. The tolerance of the precursor ions was 10 ppm and that of the fragment ions was 20 ppm. Trypsin was chosen for cleavage specificity with a maximum of two missed cleavage sites. A maximum of one O-glycosylation modification was allowed in IgA1 HR. A theoretical O-glycopeptide library was created based on the tryptic peptide from the HR (HYTNPSQDVTVPCPVPSTPPTPSPSTPPTPSPSCCHPR) with the 54 probable glycan compositions (Supplementary Table S1) (Ohyama et al., 2020). The area of the extracted ion chromatogram (XIC) of a given glycopeptide of IgA1 HR was normalized against the sum of areas of all intact IgA1 O-glycopeptides, resulting in relative quantitation. The false discovery rate (FDR) was set to ≤1% at the protein level, and the Byonic score threshold value was set to 300. All of these PSMs (peptide-spectrum matches) were examined manually and filtered using standard criteria. The PSMs were accepted if there were at least two glycan oxonium ions and at least 3 b/y or c/z ions in the peptide backbone on the PSM. The glycosylation traits of IgA1 HR were calculated using formulae shown in the Supplementary Material S1.

### Statistical Analysis

The Kolmogorov-Smirnov test was used to analyze the distribution normality of the variables. Normally distributed continuous variables were expressed as the mean ± SD. Comparisons between two groups were analyzed using *t*-test (two-tailed), whereas comparisons between three groups were performed using analysis of variance (ANOVA). Variables with non-normal distribution were expressed as median and interquartile range (IQR) and were analyzed using the Mann-Whitney U test or the Kruskal–Wallis test as appropriate. Categorical variables were presented as the number (percentage) and a chi-square test was used for the analysis. The heatmap of differential glycoforms was generated using a multi-omics data analysis tool, OmicsBean (http://www.omicsbean.cn). The diagnostic performance of each statistically significant glycosylation traits was evaluated by calculating their sensitivity and specificity using ROC curves. In addition, logistic regression analysis was performed to evaluate the diagnostic performance of the combined panel. We worked out the optimal cutoff values to maximize the sum of sensitivity and specificity, derived corresponding AUC and *p* value of each curve. A value of *p* < 0.05 was considered as statistically different. All statistics were performed using Origin software (Origin Lab Corporation, 2021), SPSS 23.0 (IBM, Chicago, IL, United States ) and GraphPad Prism 7.0.

## Results

### Clinical and Pathological Data

The characteristics of the participants were described in Table 1. The median plasma IgA and Gd-IgA1 concentrations among IgAN patients significantly were higher than those of the non-IgA nephropathy disease controls (DC) or healthy participant controls (HC) group (IgA:3.03 ± 0.86 g/L vs. 2.08 ± 0.75 vs. 1.89 ± 0.85; Gd-IgA1: 7.73 ± 3.98 μg/mL vs. 4.98 ± 3.36 vs. 5.51 ± 4.14, respectively). The estimated glomerular filtration rate (eGFR) in the IgAN group (60.02 ± 28.26 ml/kg/1.73 m^2^) was lower when compared to those of the DC (98.04 ± 37.29 ml/kg/1.73 m^2^) or HC group (120.04 ± 12.32 ml/kg/1.73 m^2^).

**TABLE 1:** Clinicopathological characteristics of participants.

Characteristics	IgAN (*n* = 62)	DC (*n* = 30)	HC(*n* = 30)	*p* value
Sex (M/F), n	32/30	18/12	16/14	0.75
Age(Y), mean ± SD	39.10 ± 11.77	44.27 ± 13.43	39.03 ± 9.25	0.11
MAP (mmHg), mean ± SD	95.94 ± 13.92	99.28 ± 12.30		0.27
Proteinuria, (g/d),mean ± SD	2.22 ± 1.61	3.07 ± 2.10		0.06
eGFR (ml/kg/1.73 m^2^), mean ± SD	60.02 ± 28.26	98.04 ± 37.29a	117.30 ± 15.53b	<**0.001**
IgA(g/L), mean ± SD	3.03 ± 0.86	2.08 ± 0.75a	1.89 ± 0.85b	<**0.001**
Gd-IgA1(μg/mL), mean ± SD	7.73 ± 3.98	4.98 ± 3.36a	5.51 ± 4.14b	<**0.001**
Oxford classification, n (%)				
M (0/1)	13(21.0)/49(79.0)			-
E (0/1)	5(8.1)/57(91.9)			-
S (0/1)	7(11.3)/55(88.7)			-
T (0/1/2)	18(29.0)/25(40.3)/19(30.7)			-
C (0/1/2)	15(24.2)/40(64.5)/7(11.3)			-
Treatment, n (%)				<**0.001**
None	20 (32.3)	3 (10.0)		
ACEI or ARB	35 (56.5)	10 (33.3)		
Steroid	7 (11.2)	17 (56.7)		

Abbreviation: ACEI: angiotensin converting enzyme inhibitor; ARB: Angiotensin receptor blocker. There were significant differences in eGFR, IgA and Gd-IgA1 levels among the three groups (*p* < 0.001). The proportions of patients who were untreated, treated with ARB or ACEI, and steroids were significantly different between the IgAN and DC groups (*p* < 0.001). *p*a < 0.05: IgAN, vs. DC; *p*b< 0.05: IgAN, vs. HC., The five pathological variables in Oxford classification[17] were scored as follow: mesangial hypercellularity ≤0.5(M0) or >0.5(M1), endocapillary hepercellularity absence(E0) or presence(E1), segmental glomerulosclerotic absence(S0) or presence(S1), tubular atrophy/interstitial fibrosis≤25% (T0) or 25–50% (T1) or >50% (T2), crescent absence(C0) or crescent presence≤25% (C1) or >25% (C2).

### Quantitative Analysis of IgA1 O-Glycopeptides Between the Three Groups

**FIGURE 3:**
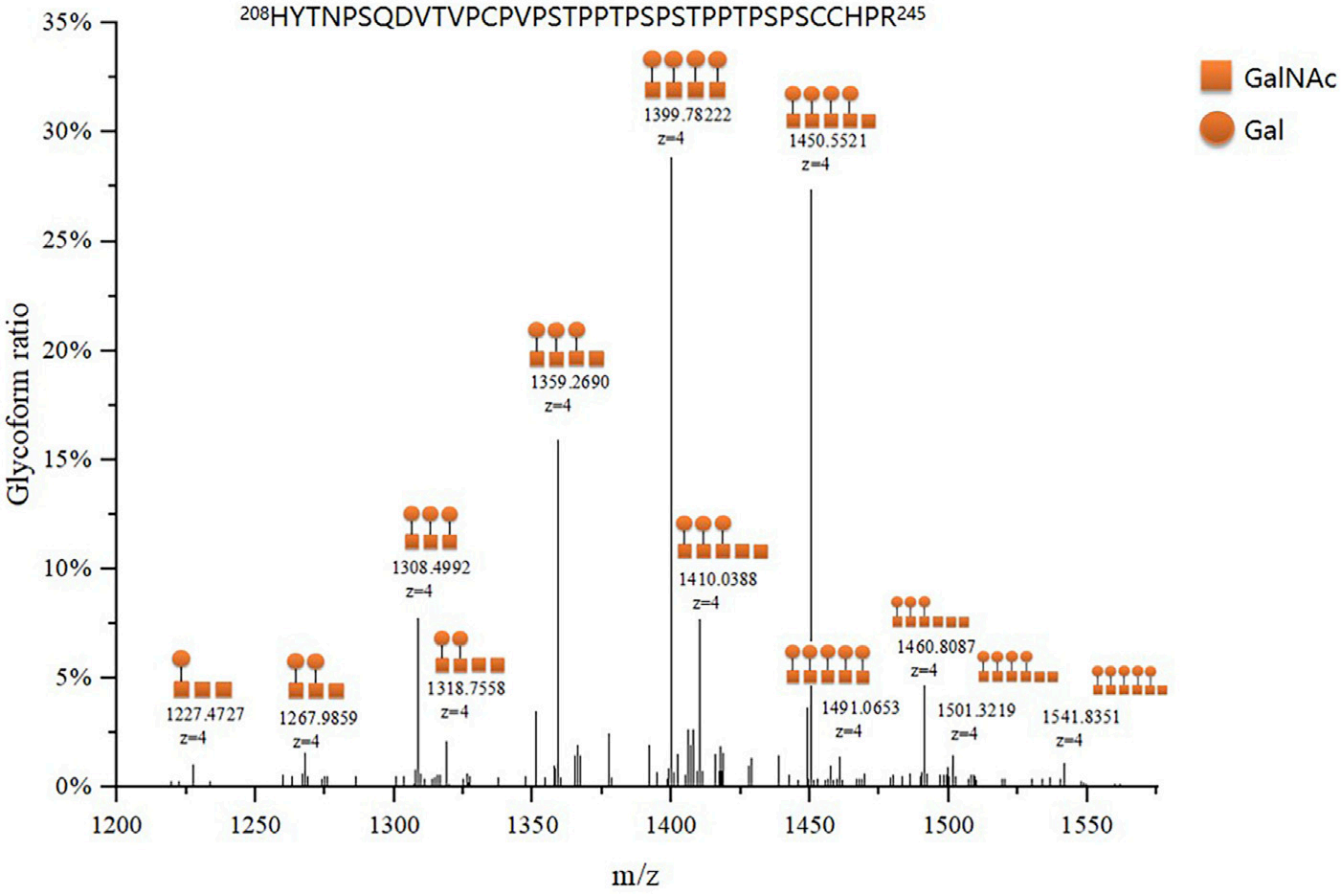
Representative mass spectrum of plasma IgA1 HR O-glycopeptides. The main twelve IgA1 HR O-glycopeptides were detected by LC-EThcD-MS/MS according to the difference in molecular weight arising from different numbers of attached monosaccharides to the peptides of IgA1 HR. Each IgA1 HR glycoform ratio was expressed as a percentage of all O-glycoforms detected.

**FIGURE 4:**
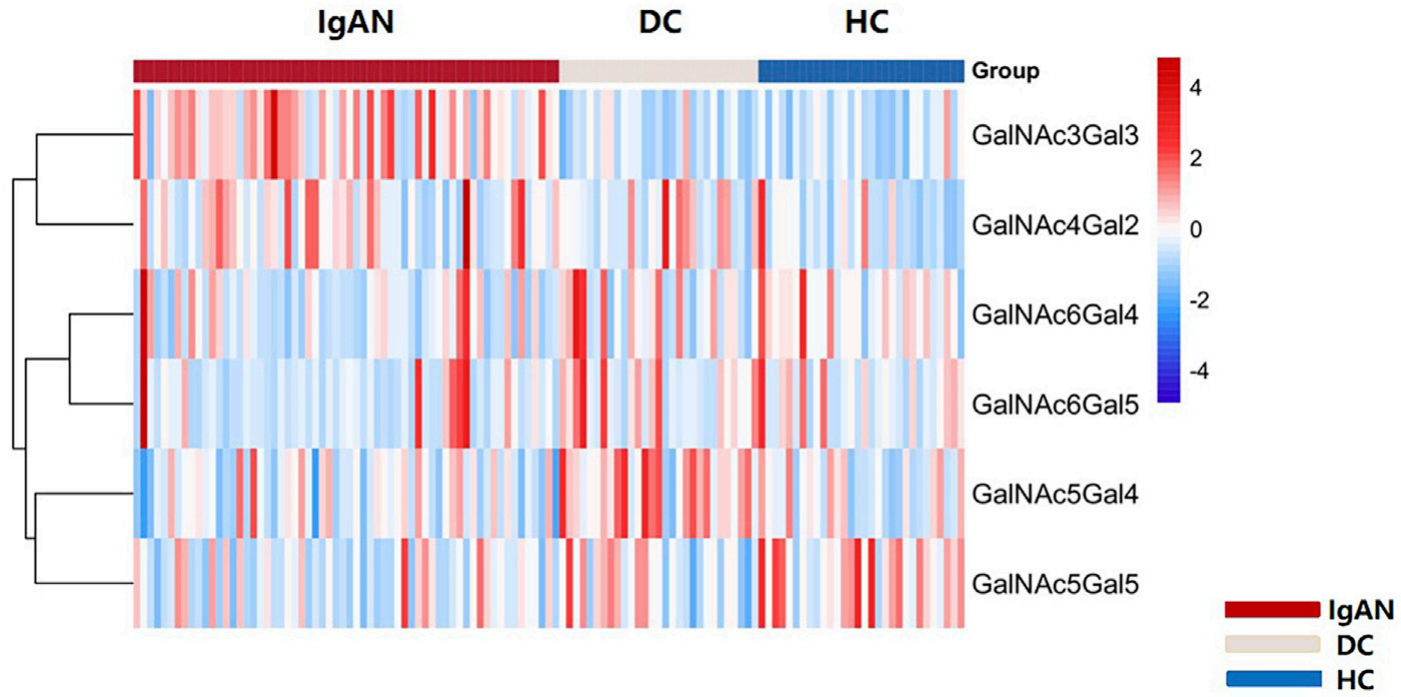
Heatmap of six differential glycoforms in IgA1 HR among IgAN, DC and HC group. The bar represented Z-score changed from-4 to 4. Similarly, the color of the bar from red to blue represented the fold change from increasing to decreasing of O-glycoforms in IgA1 HR.

We identified 48 intact IgA1 O-glycopeptides (Supplementary Figure S1) from the 122 enrolled participants, including 46 IgA1 O-glycopeptides from patients with IgAN, 42 IgA1 O-glycopeptides from DCs, and 43 IgA1 O-glycopeptides from HCs. After filling in the missing values, we derived 12 glycopeptides. Figure 3 showed the representative mass spectrum of main 12 glycopeptides in IgA1 HR detected by LC-EThcD-MS/MS. The relative abundance of each intact O-glycopeptide in plasma IgA1 was shown in Supplementary Table S2. GalNAc4Gal4 and GalNAc5Gal4 O-glycans were the predominant glycoforms observed in all three groups (Table 2). As shown in Table 2, the relative abundance of GalNAc3Gal3 in the IgAN group (7.52% [5.30–10.00%]) was significantly higher than that in the DC group (4.00% [2.76–5.30%]), while the relative abundance of GalNAc5Gal4 (27.36 ± 6.02%) and GalNAc6Gal5 (0.73% [0.43–1.00%]) in the IgAN group was significantly lower compared to those in the DC group (34.31 ± 7.37% and 1.28% [0.66–2.41%]). Heatmap analysis was performed on the relative abundance of six differential glycoforms in the IgAN group, which could be distinguished from the DC and HC groups. The glycoforms with fewer O-glycans (GalNAc3Gal3 and GalNAc4Gal2) were increased, whereas the glycoforms with more O-glycans (GalNAc5Gal4 and GalNAc5Gal5) were decreased in IgAN patients (Figure 4).

**TABLE 2:** Comparison of 12 glycoform ratios of plasma IgA1 HR in the participants.

Glycoform Ratio	IgAN (*n* = 62)	DC (*n* = 30)	HC (*n* = 30)	*p* value
GalNAc3Gal1, median (IQR)	0.38% (0.18–1.31%)	0.70% (0.09–2.71%)	0.36% (0.11–1.26%)	0.542
GalNAc3Gal2, median (IQR)	1.49% (0.81–2.09%)	1.18% (0.63–1.90%)	1.18% (0.85–1.39%)	0.144
GalNAc3Gal3, median (IQR)	7.52% (5.30–10.00%)	4.00% (2.76–5.30%)a	4.39% (3.08–5.50%)b	<**0.001**
GalNAc4Gal2, median (IQR)	1.96% (1.00–2.76%)	1.67% (1.08–2.92%)	0.98% (0.69–1.72%)b	**0.005**
GalNAc4Gal3, mean ± SD	15.86 ± 4.62%	14.08 ± 5.27%	15.11 ± 3.11%	0.206
GalNAc4Gal4, mean ± SD	28.85 ± 7.06%	29.35 ± 8.35%	29.02 ± 6.46%	0.953
GalNAc5Gal3, median (IQR)	6.83% (4.36–11.31%)	5.94% (4.23–8.53%)	6.31 (4.71–10.08%)	0.631
GalNAc5Gal4, mean ± SD	27.36 ± 6.02%	34.31% ± 7.37%a	28.43 ± 5.17%	<**0.001**
GalNAc5Gal5, median (IQR)	4.15% (2.91–6.38%)	5.41% (3.57–7.27%)	6.47% (5.26–9.20%)b	<**0.001**
GalNAc6Gal3, median (IQR)	1.03% (0.65–1.96%)	1.37% (0.58–2.71%)	1.34% (0.88–2.53%)	0.111
GalNAc6Gal4, median (IQR)	1.13% (0.75–2.11%)	1.75% (0.86–2.97%)	1.90% (1.48–2.29%)b	**0.014**
GalNAc6Gal5, median (IQR)	0.73% (0.43–1.00%)	1.28% (0.66–2.41%)a	0.90% (0.60–1.91%)	**0.006**

Abbreviation: IQR: interquartile range. The *p* value represents the statistical difference in the relative abundance of glycoforms among the three groups. *p*a < 0.05: IgAN, vs. DC; *p* b < 0.05: IgAN, vs. HC.

### Quantitative Analysis of IgA1 O-Glycosylation Traits Between the Three Groups

**FIGURE 5:**
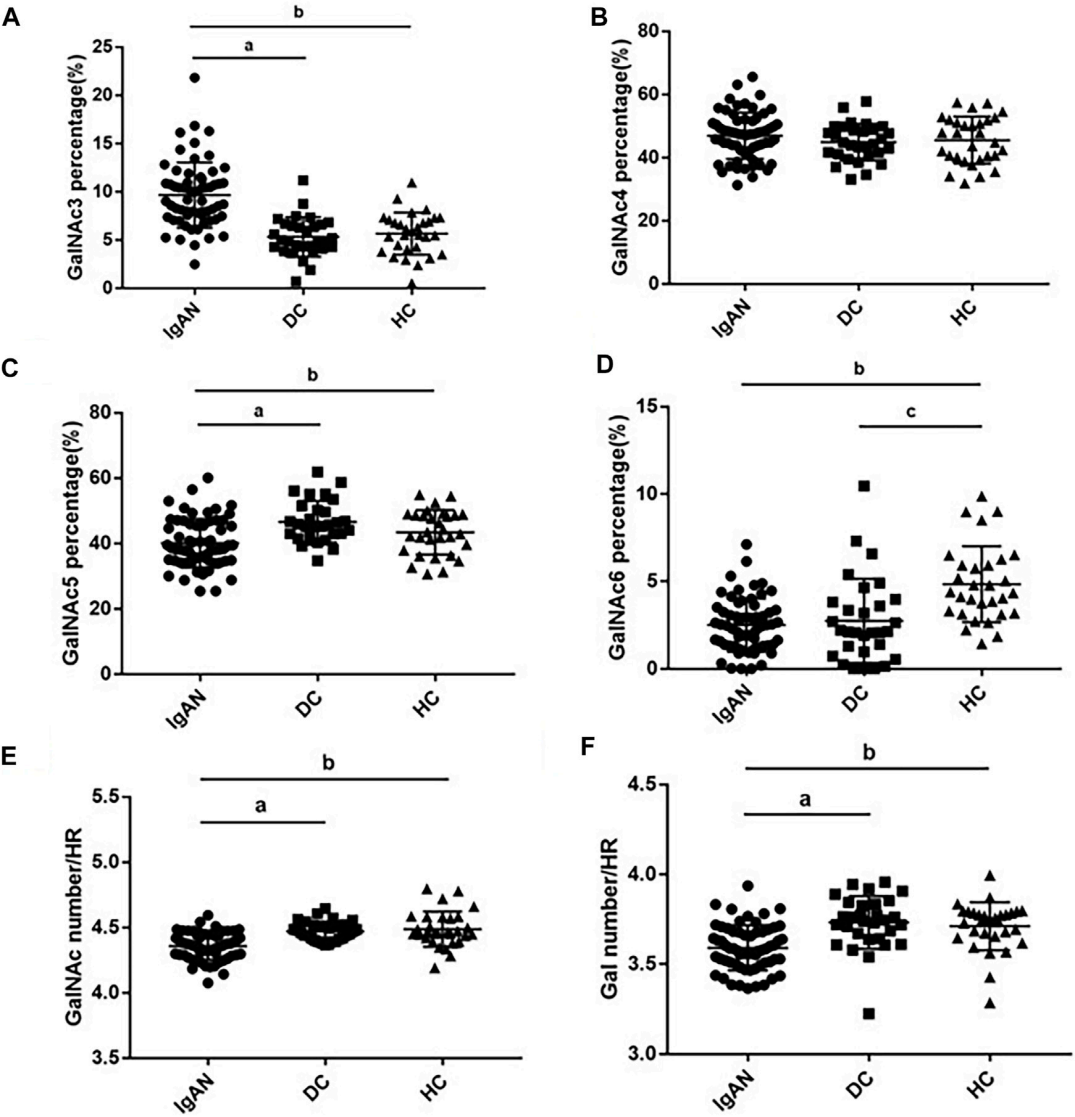
Glycosylation traits in plasma IgA1 HR among the three groups. Comparison of GalNAc3-6 ratios in plasma IgA1 HR among IgAN, DC and HC groups **(A–D)**. Comparison of Gal and GalNAc numbers in plasma IgA1 HR among IgAN, DC and HC groups **(E,F)**. *p*a < 0.05: IgAN vs. DC; *p* b < 0.05: IgAN vs. HC; *p*c<0.05: DC vs. HC.

Next, we compared the relative abundance of glycopeptides GalNAc three to six in the IgA1 HR among the three groups. The relative abundance of GalNAc3 in IgAN patients was significantly higher than that in the DC and HC groups (IgAN vs. DC: 9.65 ± 3.38% vs. 5.32 ± 2.05%; IgAN vs. HC: 9.65 ± 3.38% vs. 5.66 ± 2.20%). The relative abundance of GalNAc5 in the IgAN group was significantly lower than in the DC and HC groups (IgAN vs. DC: 40.08 ± 7.45% vs. 46.58 ± 6.37%; IgAN vs. HC: 40.08 ± 7.45% vs. 43.37 ± 6.78%). Similarly, the relative abundance of GalNAc6 was significantly reduced in the IgAN group compared with the HC group, but there was minimal difference between IgAN and the DC group (IgAN vs DC: 2.51 ± 1.52% vs. 2.75 ± 2.41%; IgAN vs. HC: 2.51 ± 1.52% vs. 4.84 ± 2.17%). There was no significant statistical difference in the relative abundance of GalNAc4 between the three groups (Figures 5A–D).

The number of GalNAc in the plasma IgA1 HR in patients with IgAN was significantly lower than that in the DC and HC groups (IgAN vs DC vs. HC: 4.36 ± 0.10 vs. 4.47 ± 0.07 vs. 4.49 ± 0.14). Similar results were found for the number of Gal (IgAN vs. DC vs. HC: 3.59 ± 0.13 vs. 3.73 ± 0.15 vs. 3.71 ± 0.13) (Figures 5E, F).

### Diagnostic Performance of IgA1 O-Glycosylation Characteristics

**FIGURE 6:**
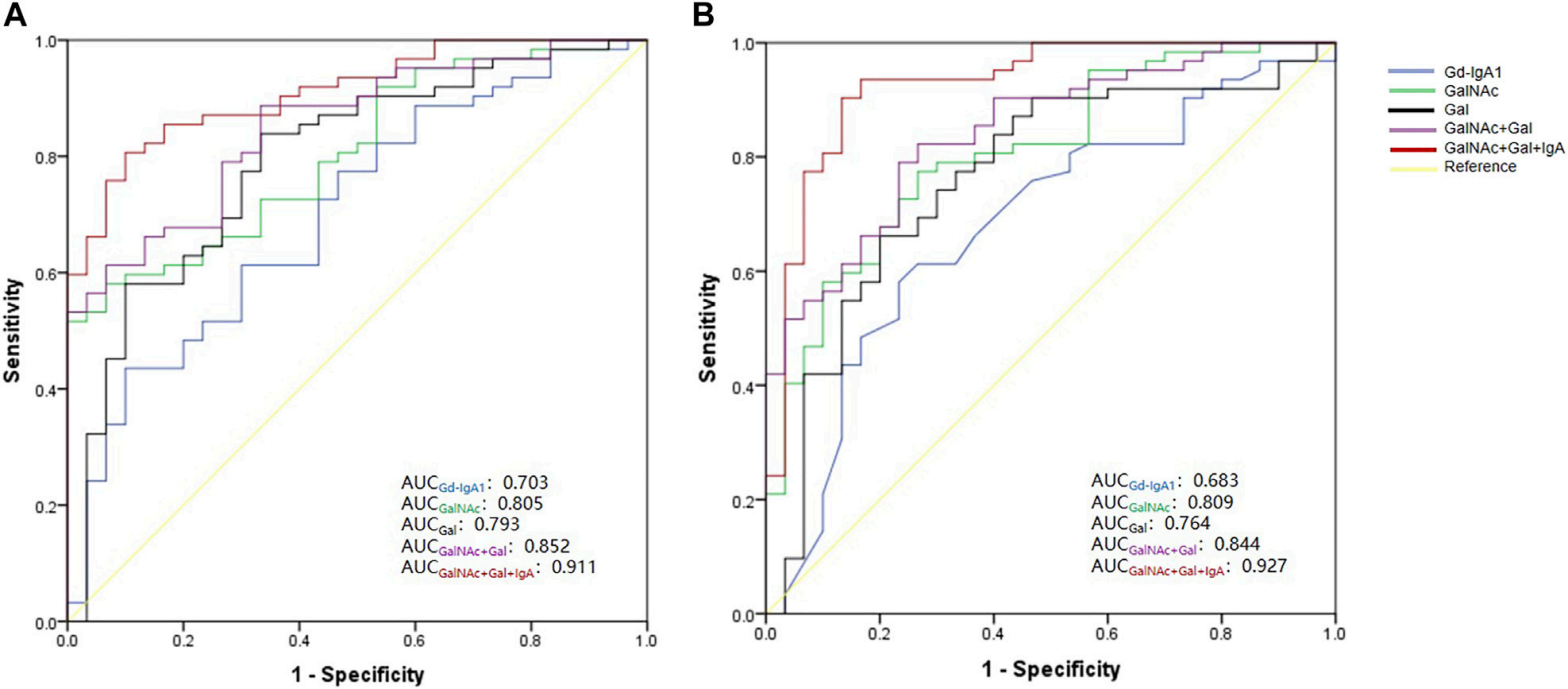
ROC curve for five biomarkers—Gd-IgA1, GalNAc number, Gal number, combined GalNAc-Gal and GalNAc-Gal-IgA Panel for the diagnosis of IgAN. **(A)** ROC curve analysis between IgAN and DC group. The AUC for GalNAc-Gal-IgA Panel was higher than those for Gd-IgA1, GalNAc, Gal and GalNAc-Gal (GalNAc-Gal-IgA panel vs. combined Gal and GalNAc, *p* = 0.029; GalNAc-Gal-IgA panel vs. GalNAc, *p* = 0.004; GalNAc-Gal-IgA panel vs. Gal, *p* = 0.016; GalNAc-Gal-IgA panel vs. Gd-IgA1, *p* < 0.001). **(B)** ROC curve analysis between IgAN and HC group. The AUC for GalNAc-Gal-IgA Panel was higher than those for Gd-IgA1, GalNAc, Gal and GalNAc-Gal (GalNAc-Gal-IgA panel vs. combined Gal and GalNAc, *p* = 0.027; GalNAc-Gal-IgA panel vs. GalNAc, *p* = 0.006; GalNAc-Gal-IgA panel vs. Gal, *p* = 0.006; GalNAc-Gal-IgA panel vs. Gd-IgA1, *p* < 0.001).

We performed receiver operating characteristic (ROC) curve analysis and calculated the area under the curve (AUC) of the plasma Gd-IgA1 levels and numbers of GalNAc and Gal of IgA1 HRs for the diagnosis of IgAN (Figure 6, Supplementary Table S3). Our results showed that comparing the DC and HC groups, the AUC values of Gd-IgA1 level in diagnosing IgAN were 0.703 (95% CI: 0.591–0.815) and 0.683 (95% CI: 0.565–0.802) respectively. Compared with DC and HC, the AUC values of Gal, GalNAc, and the combination of Gal and GalNAc in diagnosing IgAN were significantly higher than those of plasma Gd-IgA1 levels (Gal, GalNAc, GalNAc-Gal panel for DC: 0.793 vs 0.805 vs 0.852; Gal, GalNAc, GalNAc-Gal panel for HC: 0.764 vs. 0.809 vs. 0.844). We further combined plasma IgA level with IgA1 O-glycosylation traits (combined Gal and GalNAc) to form a panel for IgAN diagnosis. The GalNAc-Gal-IgA panel had the strongest diagnostic performance among all the above five markers for distinguishing IgAN from DC and HC participants, with AUC values of 0.911 (95% CI: 0.854–0.967) the DC group and 0.927 (95% CI: 0.866–0.988) when compared with the HC group.

## Discussion

In this study, we used IgA immunoaffinity beads to extract plasma IgA and performed LC-EthCD-MS/MS to quantitatively detect differences in IgA1 HR O-glycopeptides and O-glycosylation traits in patients with IgAN, patients with non-IgA glomerulonephropathy, and healthy participants. The results suggested that, compared with the DC and HC groups, the GalNAc and Gal numbers of plasma IgA1 HR in IgAN patients were significantly reduced. The combination of GalNAc and Gal numbers had a better diagnostic performance than Gd-IgA1 level measured by KM55. The GalNAc-Gal-IgA panel in this study had a high performance for diagnosing IgAN and is expected to be a non-invasive biomarker replacing renal biopsy.

Proteomic studies have found that Gal and SA modifications of the circulating IgA1 HRs in patients with IgAN were deficient. Two recent studies (Nakazawa et al., 2019a; Nakazawa et al., 2019b) analyzed O-glycans of circulating IgA1 HRs using matrix-assisted laser desorption/ionization time-of-flight (MALDI-TOF) mass spectrometry and found that the number of GalNAc and Gal in patients with IgAN was lower than those in healthy participants. To the best of our knowledge, few studies have detected the O-glycosylation patterns of IgA1 HRs using high-resolution mass spectrometry technology to improve IgAN diagnosis. In this study, we developed a novel diagnostic tool, and found that the AUC level of the GalNAc-Gal-IgA panel for distinguishing IgAN from DC and HC were 0.911 and 0.927, respectively, which was better than the Gd-IgA1 levels measured by KM55 and other individual O-glycosylation traits. Except for serum Gd-IgA1, biomarkers currently studied for IgAN diagnosis performance evaluation include the IgA1-alpha-1-microglobulin complex and sCD89-IgA complex. The study (Xu et al., 2021) found that the blood IgA1-alpha-1-microglobulin complex could be a potential biomarker in IgAN, with an AUC of 0.862, which was also better than that of serum Gd-IgA1 (AUC of 0.732) in the same cohort, which was similar to our results. Another study (Wu et al., 2020) found that, compared with healthy controls, the AUC of serum sCD89-IgA for diagnosing IgAN was 0.762.

Some researchers have examined O-glycoproteomes in human serum (Zhang et al., 2018) using Byonic, a glycopeptide quantitative software. We used it to automatically and manually analyze the O-glycopeptides of the 122 individuals. Recently, a study (Dotz et al., 2021) used IgA immunoaffinity beads to enrich IgA from the blood of patients with IgAN and healthy controls to quantify 69 O- and N-glycosylation traits. This study also found that directly measured glycopeptide-level IgA glycosylation traits were more determinable for IgAN status and disease characteristics than Gd-IgA levels.

The analysis of the correlations between plasma Gd-IgA1 levels and the numbers of GalNAc and Gal in IgAN patients showed that the number of Gal was significantly negatively correlated with the plasma Gd-IgA1 level (Supplementary Table S4), which was consistent with the result of (Dotz et al., 2021). As the GalNAc number decreased, the level of plasma IgA1 increased, although the association was not statistically significant. KM55 was obtained by immunizing rats with a synthetic IgA1 HR peptide with GalNAc residues, and KM55 was able to recognize naked GalNAc residues (Yasutake et al., 2015). It is necessary to evaluate the associations between circulating Gd-IgA1 levels and the numbers of GalNAc and Gal in IgAN cohort with a large sample size. We firstly found that the number of Gal in the IgA1 HR was positively correlated with the level of serum C3 (Supplementary Table S4), suggesting that the severe deficiency of the number of Gal in the HR of IgA1 may related to the activation of the alternative pathway of complement in IgAN patients. Whereas IgAN is a complement-mediated disease in which alternative and lectin pathway of complements are activated (Le Stang et al., 2021).

We did not find correlations between the numbers of GalNAc and Gal and eGFR in the IgAN group (Supplementary Table S4), which is consistent with the results recently reported by (Yu et al., 2021). Another study (Dotz et al., 2021) showed only significantly positive association of the sialylation with eGFR. We cannot demonstrate the correlation between sialylation modifications and eGFR since desialylation was performed in our study. Yu et al. (2021) found that the number of GalNAc in the IgA1 HR in severe crescentic IgAN patients (crescents in >50% of the glomeruli) was less than those of the IgAN patients with crescents in 0–50% of the glomeruli and healthy controls. Different from the cohort of Yu G et al., our cohort did not have severe crescentic IgAN patients (crescent presence >50%), and we divided patients into C0 (crescent absence), C1 (crescent presence ≤25%) and C2 (crescent presence >25%) groups according to the Oxford classification, no difference in the numbers of GalNAc and Gal was found among 3 C score groups. However, 64.5% of our patients were scored C1, unlike socre of C2, a score of C1 predicts a poor renal outcome only in patients without receiving immunosuppressive therapy (Trimarchi et al., 2017), and most of the patients in group of C1 received steroid therapy in our cohort. Similarly, we did not find significantly differences of the numbers of GalNAc and Gal in the IgA1 HR among groups with different MEST T scores (T0 ≤25%, T1 25–50% and T2 ≥50%). As our knowledge, there is no report on the association of T scores with the numbers of GalNAc and Gal in the IgA1 HR.

A limitation of this study was that desialylation was carried out with reference to previous studies (Inoue et al., 2012; Nakazawa et al., 2019a; Nakazawa et al., 2019b), which reduced the difficulty of searching the O-glycopeptide library but caused a lack of information on SA in the IgA1 HR. However, this did not affect the accuracy of the remaining glycoform identification. Another limitation is the lack of validation in the study. There were few patients with mild proteinuria and/or isolated microscopic hematuria hospitalized for renal biopsy due to the policy of renal biopsy in our hospital, which reduced the representativeness of the enrolled participants. It is necessary to validate the diagnostic efficacy of the GalNAc-Gal-IgA panel for IgAN in an independent multicenter cohort with fully representative IgAN patients and further explore the predictive performance of IgA glycosylation traits on the prognosis of IgAN.

Our findings expand our current understanding of the role of plasma IgA1 O-glycosylation in IgAN pathogenesis. The present glycoproteomic study applied the LC-EthCD-MS/MS strategy to analyze O-glycosylation in circulating IgA1 HR of IgAN, non-IgA glomerulonephropathy, and healthy individuals. We found that the GalNAc-Gal-IgA panel had a significantly improved diagnostic efficiency for IgAN. To our knowledge, this panel is the best circulating biomarker of IgAN. Since renal biopsy is an invasive procedure, patients are inclined to be reluctant to have the procedure performed. This diagnostic panel is expected to be a non-invasive MS-based biomarker for IgAN that can be applied in clinical practice in the future.

## Conclusion

This is the first study to explore plasma IgA1 O-glycosylation traits in order to establish diagnostic biomarkers for IgAN. The panel containing GalNAc, Gal, and circulating IgA showed excellent diagnostic performance and is promising for application in clinical practice.

## Data Availability

The datasets presented in this study can be found in online repositories. The names of the repository/repositories and accession number(s) can be found in the article/Supplementary Material.
