# Supplementary material for: Diagnostic Potential of Plasma IgA1 O-Glycans in Discriminating IgA Nephropathy From Other Glomerular Diseases and Healthy Participants
Source: Front Mol Biosci. 2022 Apr 4;9:871615. doi: 10.3389/fmolb.2022.871615 (PMC9014244; doi:10.3389/fmolb.2022.871615)

**Supplemental Figure S1.** The MS2 spectrum of intact 48 detected O-glycopepetides in IgA1 HR. The oxonium ions, glycosidic fragments, and b/y, c/z fragments from the peptide backbone were well characterized. Abbreviation: XIC: extracted ion chromatogram.

1. **The MS2 spectrum of GalNAc1 glycopeptide in IgA1 HR.**

GalNAc1 m/z=1085.7518 z＝4 Retention Time (min) = 21.42

XIC Area = 3.19e+7 score=731.67 Error (ppm) = 3.66


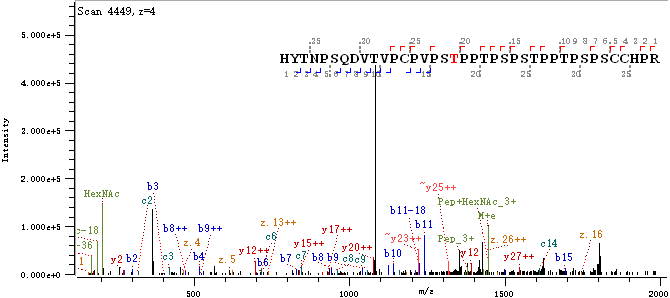


1. **The MS2 spectrum of GalNAc1Gal1 glycopeptide in IgA1 HR.**

GalNAc1Gal1 m/z=901.2143 z＝5 Retention Time (min)＝ 21.77

XIC Area = 2.46e+7 score=752.81 Error (ppm) = 4.46


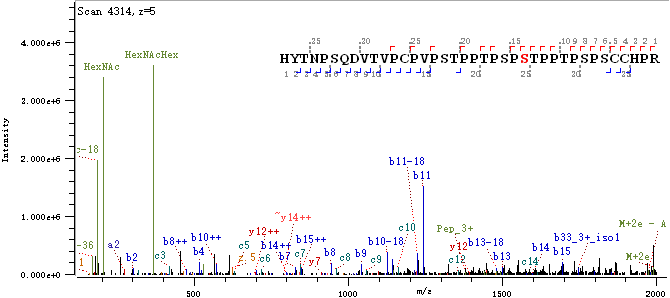


1. **The MS2 spectrum of GalNAc2 glycopeptide in IgA1 HR.**

GalNAc2  m/z=1515.0085 z＝3 Retention Time (min)＝19.68

XIC Area = 6.82e+6 score=388.99 Error (ppm) = -8.30


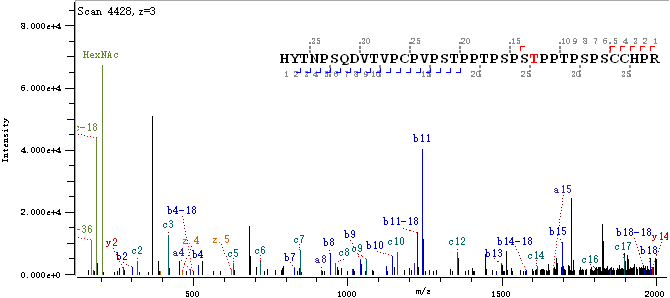


1. **The MS2 spectrum of GalNAc2Gal1 glycopeptide in IgA1 HR.**

GalNAc2Gal1 m/z=1177.0296 z＝4 Retention Time (min)＝20.82

XIC Area = 1.46e+8 score=759.88 Error (ppm) =-1.06


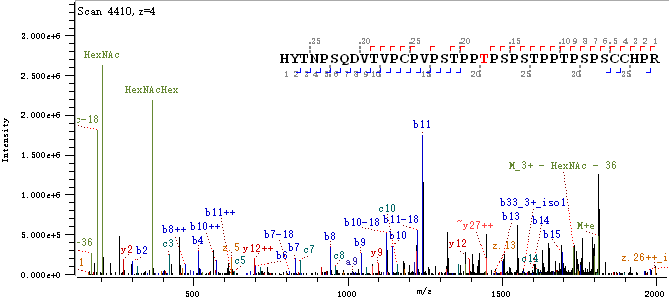


1. **The MS2 spectrum of GalNAc2Gal2 glycopeptide in IgA1 HR.**

GalNAc2Gal2 m/z= 1221.5339 z＝4 Retention Time (min)＝22.46

XIC Area =1.37e+7 score=499.06 Error (ppm) =-7.26


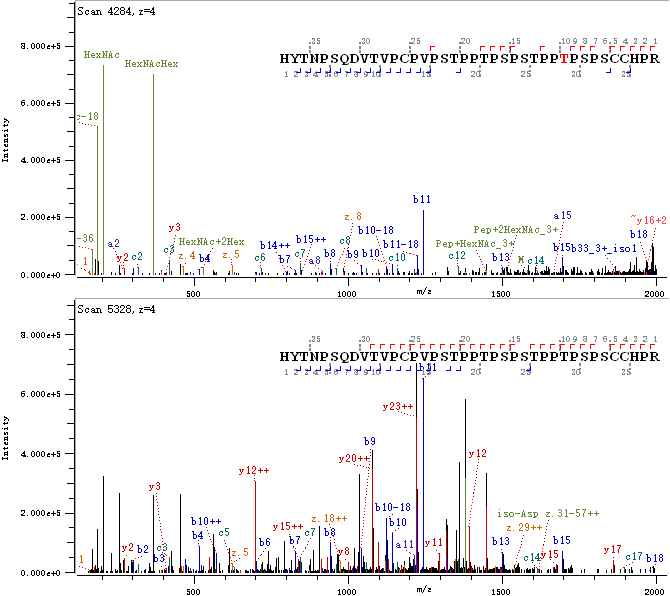


1. **The MS2 spectrum of GalNAc3 glycopeptide in IgA1 HR.**

GalNAc3 m/z=1582.7024 z＝3 Retention Time (min)＝22.44

XIC Area =6.92e+7 score=531.07 Error (ppm) =-7.43


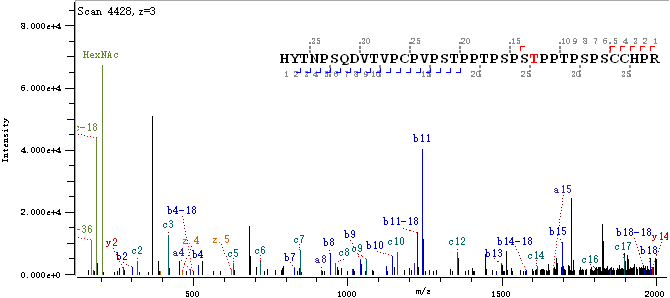


1. **The MS2 spectrum of GalNAc3Gal1 glycopeptide in IgA1 HR.**

GalNAc3Gal1 m/z=982.4461 z＝5 Retention Time (min)＝21.11

XIC Area =2.48e+7 score=781.16 Error (ppm) =4.17


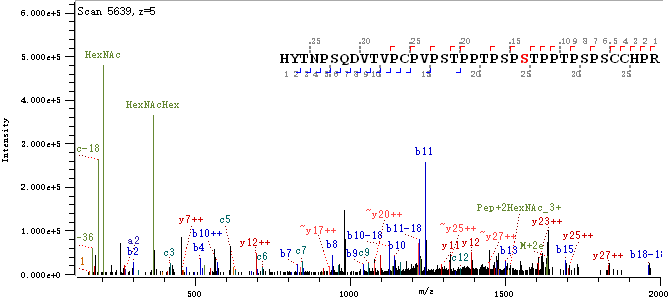


1. **The MS2 spectrum of GalNAc3Gal2 glycopeptide in IgA1 HR.**

GalNAc3Gal2 m/z=1014.8524 z＝5 Retention Time (min)＝19.74

XIC Area =4.69e+8 score=977.74 Error (ppm) = -0.14


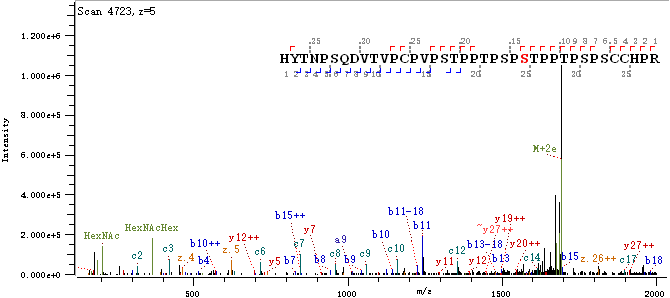


**9. The MS2 spectrum of GalNAc3Gal3 glycopeptide in IgA1 HR.**

GalNAc3Gal3 m/z=748.3337 z＝7 Retention Time (min)＝20.47

XIC Area =2.51e+7 score=446.21 Error (ppm) =1.11


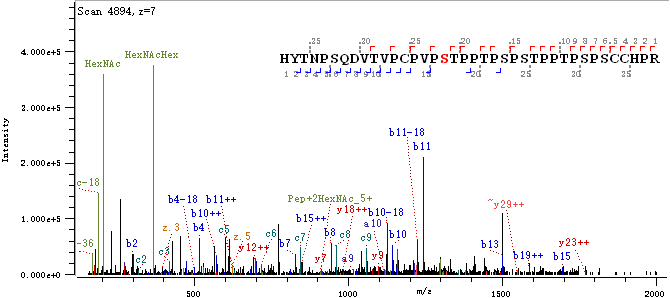


**10. The MS2 spectrum of GalNAc4 glycopeptide in IgA1 HR.**

GalNAc4 m/z=1238.0503 z＝4 Retention Time (min)＝22.06

XIC Area =2.03e+8 score=542.27 Error (ppm) =-5.67


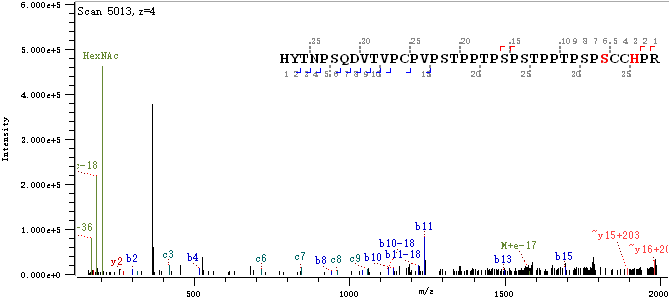


1. **The MS2 spectrum of GalNAc4Gal1 glycopeptide in IgA1 HR.**

GalNAc4Gal1 m/z=1282.5656 z＝4 Retention Time (min)＝19.53

XIC Area =9.26e+6 score=332.54 Error (ppm) = -2.87


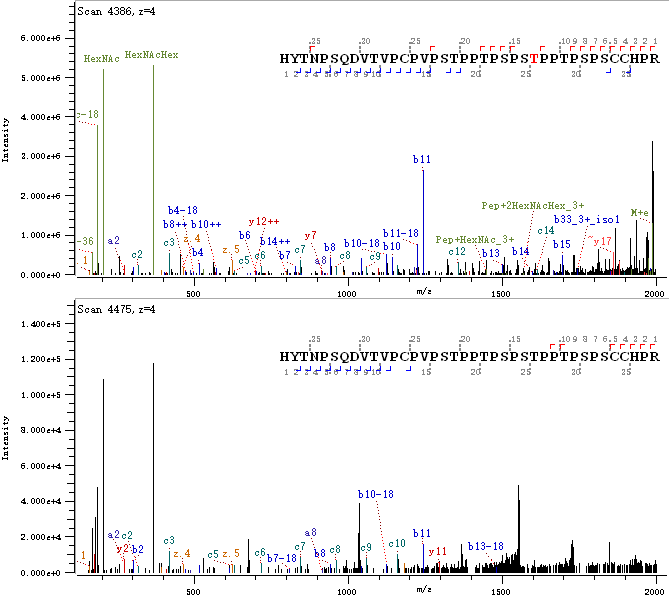


1. **The MS2 spectrum of GalNAc4Gal2 glycopeptide in IgA1 HR.**

GalNAc4Gal2 m/z=1055.4686 z＝5 Retention Time (min)＝20.00

XIC Area =3.05e+8 score=801.22 Error (ppm) = 0.19


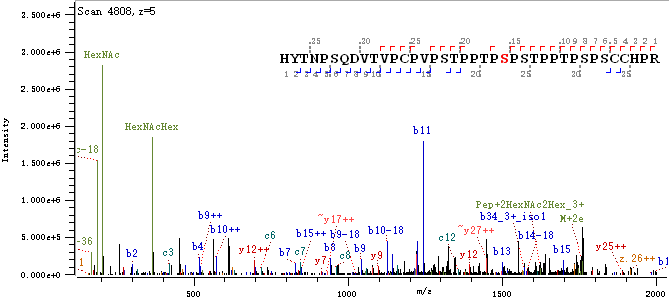


1. **The MS2 spectrum of GalNAc4Gal3 glycopeptide in IgA1 HR.**

GalNAc4Gal3 m/z=906.7346 z＝6 Retention Time (min)＝18.26

XIC Area =1.49e+8 score=372.95 Error (ppm) =1.00


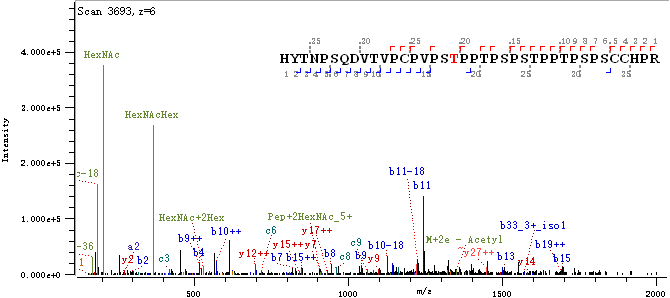


1. **The MS2 spectrum of GalNAc4Gal4 glycopeptide in IgA1 HR.**

GalNAc4Gal4 m/z=1400.1065 z＝4 Retention Time (min)＝18.99

XIC Area =8.34e+9 score=316.17 Error (ppm) =-0.96


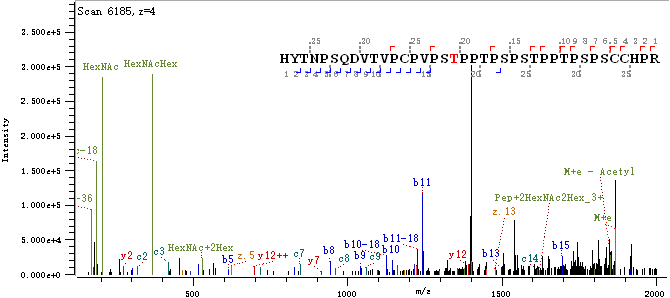


1. **The MS2 spectrum of GalNAc5 glycopeptide in IgA1 HR.**

GalNAc5 m/z=1112.4896 z＝5 Retention Time (min)＝21.08

XIC Area =7.82e+7 score=386.42 Error (ppm) =-4.77


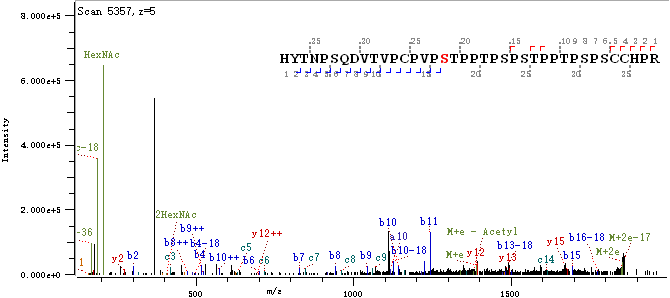


1. **The MS2 spectrum of GalNAc5Gal1 glycopeptide in IgA1 HR.**

GalNAc5Gal1 m/z=1329.3313 z＝4 Retention Time (min)＝21.37

XIC Area =1.15e+7 score=442.09 Error (ppm) = -6.83


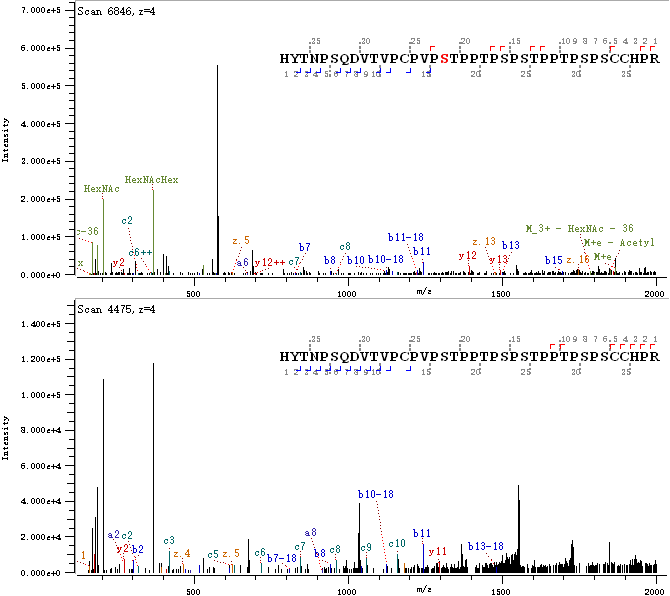


1. **The MS2 spectrum of GalNAc5Gal2 glycopeptide in IgA1 HR.**

GalNAc5Gal2 m/z=1096.0894 z＝5 Retention Time (min)＝23.23

XIC Area = 4.17e+7 score=637.82 Error (ppm) =4.63


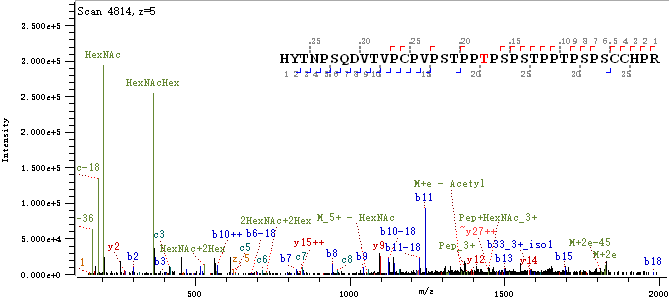


1. **The MS2 spectrum of GalNAc5Gal3 glycopeptide in IgA1 HR.**

GalNAc5Gal3 m/z=1131.6896 z＝5 Retention Time (min)＝19.74

XIC Area =1.07e+8 score=379.70 Error (ppm) = -3.78


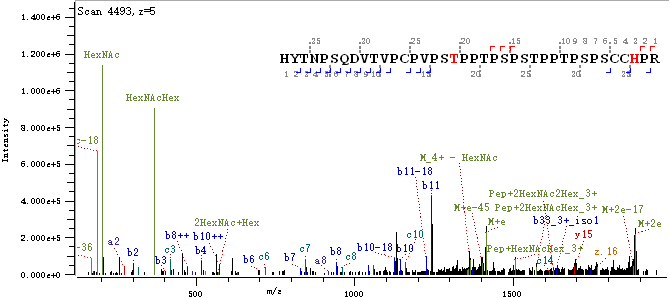


1. **The MS2 spectrum of GalNAc5Gal4 glycopeptide in IgA1 HR.**

GalNAc5Gal4 m/z=1160.9063 z＝5 Retention Time (min)＝19.13

XIC Area =1.07e+9 score=682.35 Error (ppm) =2.70


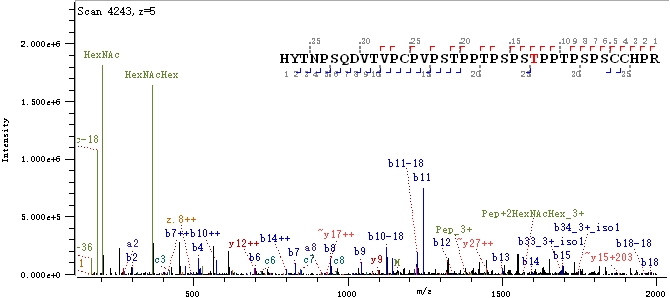


1. **The MS2 spectrum of GalNAc5Gal5 glycopeptide in IgA1 HR.**

GalNAc5Gal5 m/z=994.5997 z＝6 Retention Time (min)＝18.64

XIC Area =1.76e+8 score=365.54 Error (ppm) =1.78


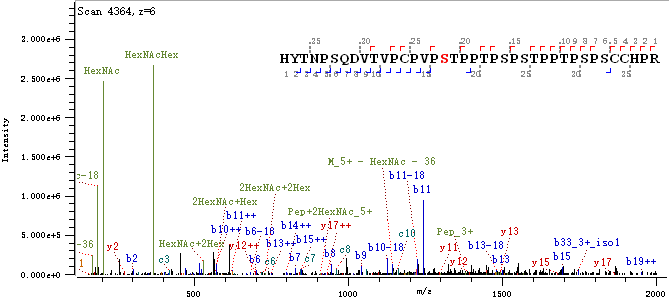


1. **The MS2 spectrum of GalNAc6 glycopeptide in IgA1 HR.**

GalNAc6 m/z=1339.5845 z＝4 Retention Time (min)＝20.35

XIC Area =4.33e+7 score=459.25 Error (ppm) =-9.33


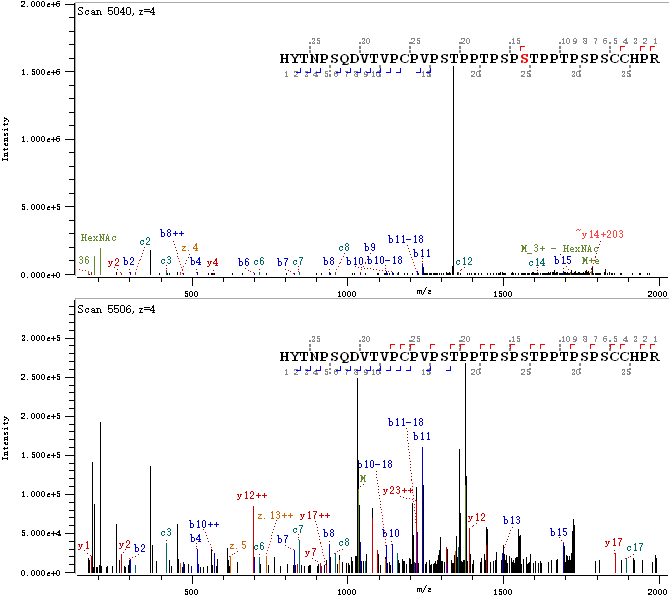


1. **The MS2 spectrum of GalNAc6Gal1 glycopeptide in IgA1 HR.**

GalNAc6Gal1 m/z=1380.1041 z＝4 Retention Time (min)＝17.78

XIC Area =8.67e+6 score=816.54 Error (ppm) =-4.40


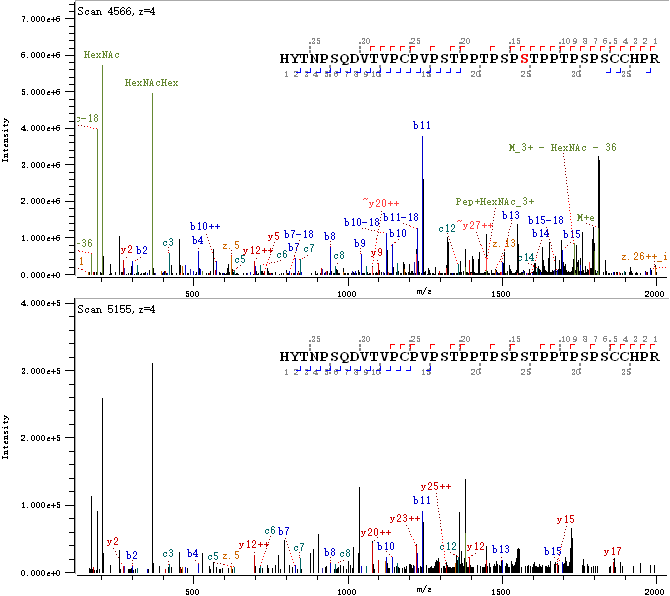


1. **The MS2 spectrum of GalNAc6Gal2 glycopeptide in IgA1 HR.**

GalNAc6Gal2 m/z=1420.6262 z＝4 Retention Time (min)＝19.38

XIC Area =6.19e+8 score=820.31 Error (ppm) =1.99


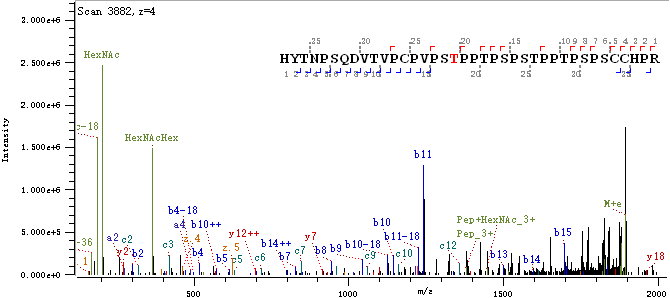


1. **The MS2 spectrum of GalNAc6Gal3 glycopeptide in IgA1 HR.**

GalNAc6Gal3 m/z=1461.1284 z＝4 Retention Time (min)＝19.43

XIC Area =4.87e+8 score=591.39 Error (ppm) = -5.60


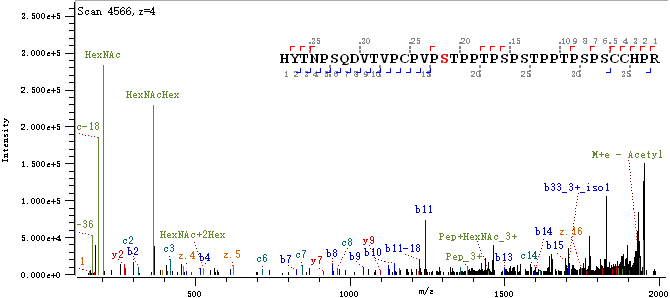


1. **The MS2 spectrum of GalNAc6Gal4 glycopeptide in IgA1 HR.**

GalNAc6Gal4 m/z=1501.6498 z＝4 Retention Time (min)＝18.67

XIC Area =5.55e+8 score=742.98 Error (ppm) =-1.08


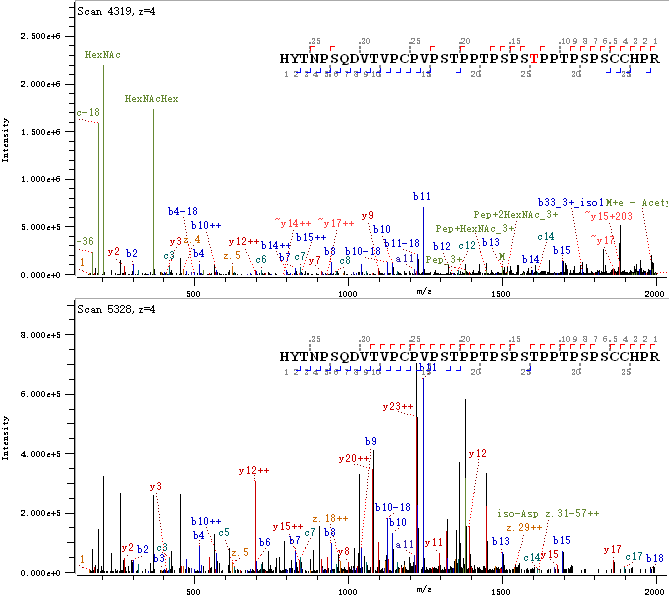


1. **The MS2 spectrum of GalNAc6Gal5 glycopeptide in IgA1 HR.**

GalNAc6Gal5 m/z=1546.1664 z＝4 Retention Time (min)＝18.91

XIC Area =1.41e+8 score=363.62 Error (ppm) = 2.99


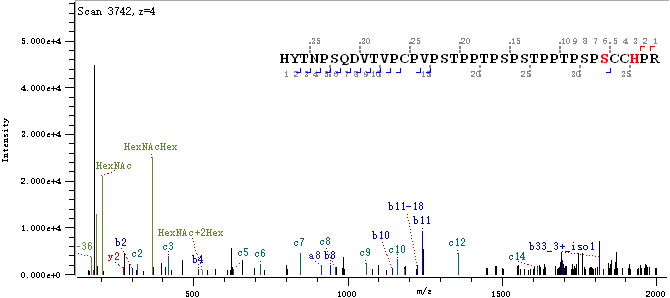


1. **The MS2 spectrum of GalNAc6Gal6 glycopeptide in IgA1 HR.**

GalNAc6Gal6 m/z=1269.5459 z＝5 Retention Time (min)＝19.26

XIC Area = 4.82e+7 score=479.70 Error (ppm) =3.53


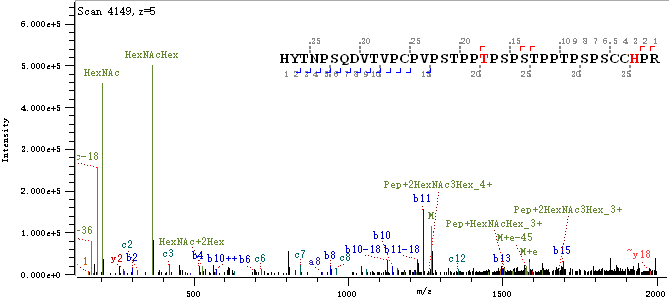


1. **The MS2 spectrum of GalNAc7 glycopeptide in IgA1 HR.**

GalNAc7 m/z=1112.4923 z＝5 Retention Time (min)＝20.07

XIC Area =8.92e+7 score=434.67 Error (ppm) = -2.36


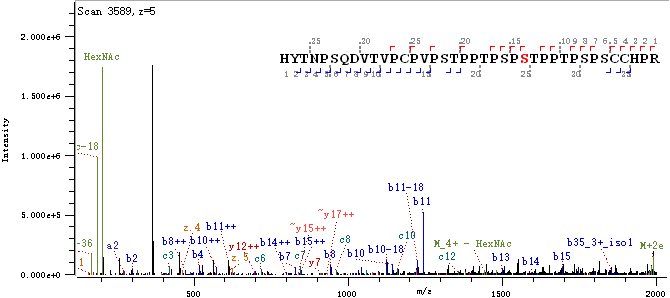


1. **The MS2 spectrum of GalNAc7Gal1 glycopeptide in IgA1 HR.**

GalNAc7Gal1 m/z=1144.9044 z＝5 Retention Time (min)＝19.45

XIC Area =3.15e+8 score=574.18 Error (ppm) = -0.93


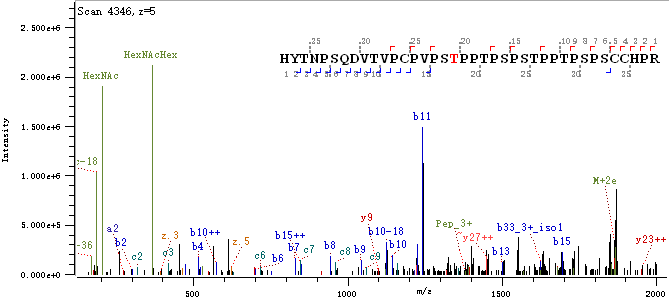


1. **The MS2 spectrum of GalNAc7Gal2 glycopeptide in IgA1 HR.**

GalNAc7Gal2 m/z=1471.3786 z＝4 Retention Time (min)＝20.36

XIC Area =3.85e+7 score=376.02 Error (ppm) = -9.99


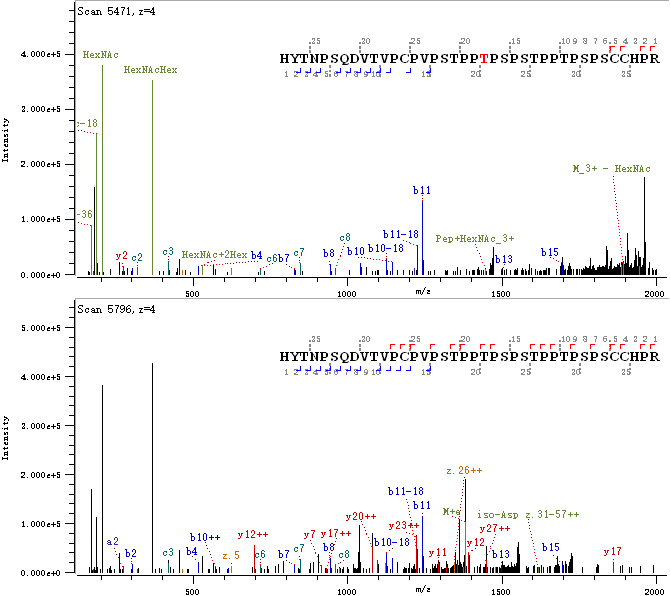


1. **The MS2 spectrum of GalNAc7Gal3 glycopeptide in IgA1 HR.**

GalNAc7Gal3 m/z=1511.8973 z＝4 Retention Time (min)＝23.07

XIC Area =5.19e+7 score=309.67 Error (ppm) =-6.03


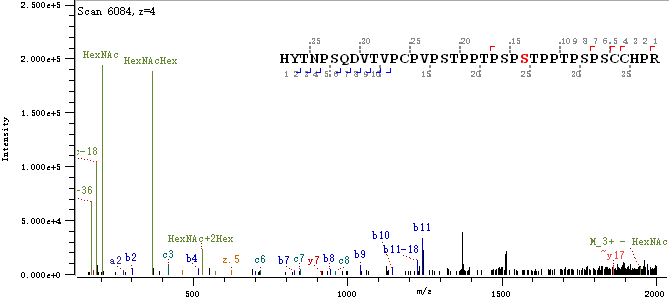


1. **The MS2 spectrum of GalNAc7Gal4 glycopeptide in IgA1 HR.**

GalNAc7Gal4 m/z=1242.1471 z＝5 Retention Time (min)＝19.59

XIC Area =3.99e+7 score=573.64 Error (ppm) =7.98


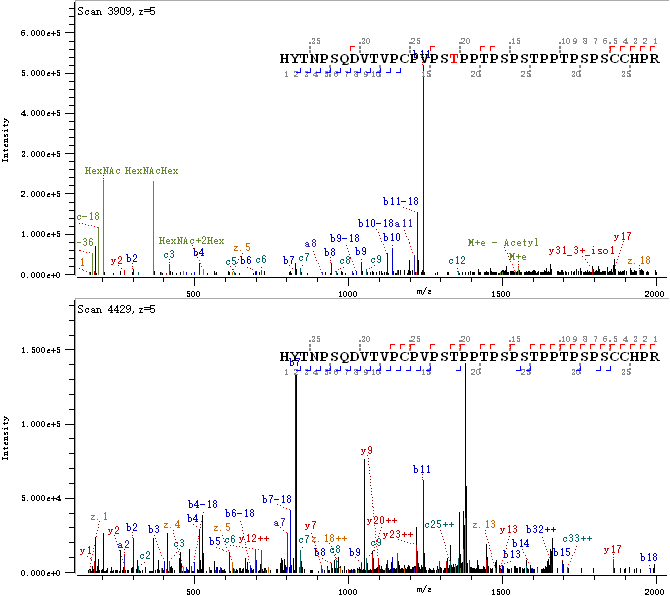


1. **The MS2 spectrum of GalNAc7Gal5 glycopeptide in IgA1 HR.**

GalNAc7Gal5 m/z=1277.7554 z＝5 Retention Time (min)＝19.17

XIC Area =1.53e+7 score=516.78 Error (ppm) =6.77


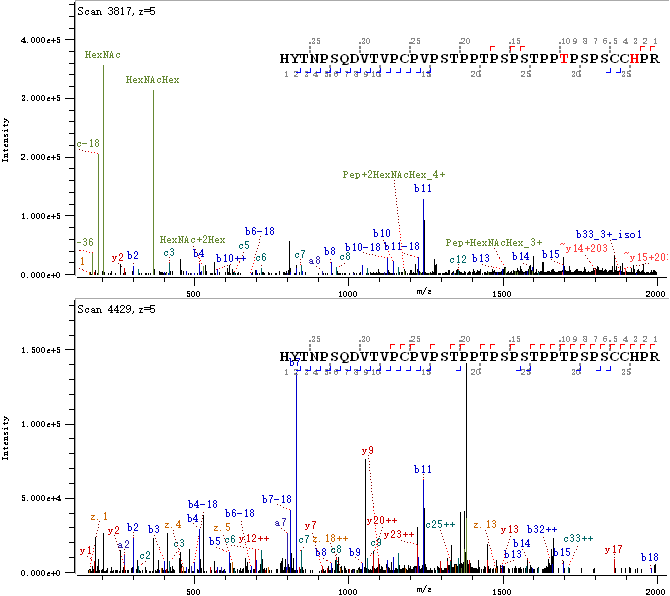


1. **The MS2 spectrum of GalNAc7Gal6 glycopeptide in IgA1 HR.**

GalNAc7Gal6 m/z=1310.1615 z＝5 Retention Time (min)＝18.80

XIC Area =4.06e+7 score=521.46 Error (ppm) =3.22


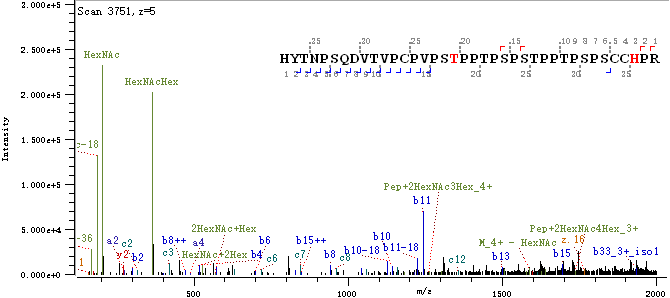


1. **The MS2 spectrum of GalNAc7Gal7 glycopeptide in IgA1 HR.**

GalNAc7Gal7 m/z=1309.9857 z＝5 Retention Time (min)＝19.38

XIC Area =3.32e+8 score=482.60 Error (ppm) =-5.08


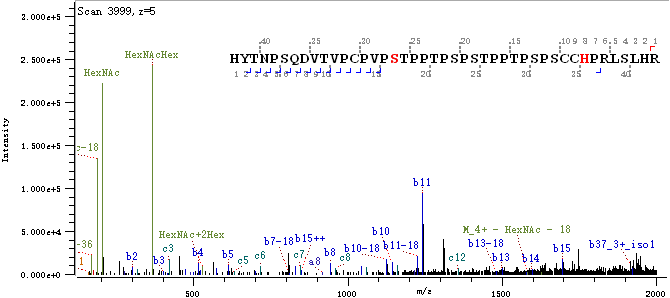


1. **The MS2 spectrum of GalNAc8 glycopeptide in IgA1 HR.**

GalNAc8 m/z=1441.1320 z＝4 Retention Time (min)＝19.85

XIC Area = 3.25e+7 score=550.13 Error (ppm) =-3.25


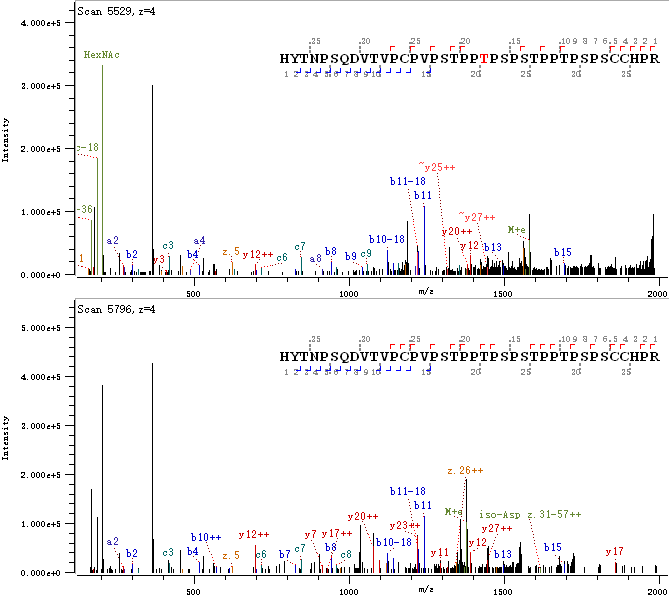


1. **The MS2 spectrum of GalNAc8Gal1 glycopeptide in IgA1 HR.**

GalNAc8Gal1 m/z=1188.7118 z＝5 Retention Time (min)＝21.55

XIC Area =1.16e+7 score=462.51 Error (ppm) = -7.19


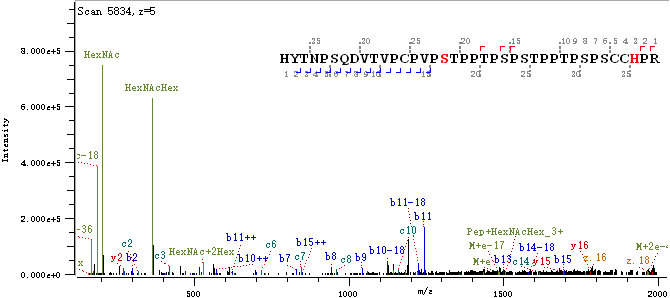


1. **The MS2 spectrum of GalNAc8Gal2 glycopeptide in IgA1 HR.**

GalNAc8Gal2 m/z=1522.1525 z＝4 Retention Time (min)＝18.49

XIC Area =2.54e+7 score=524.48 Error (ppm) = -6.99


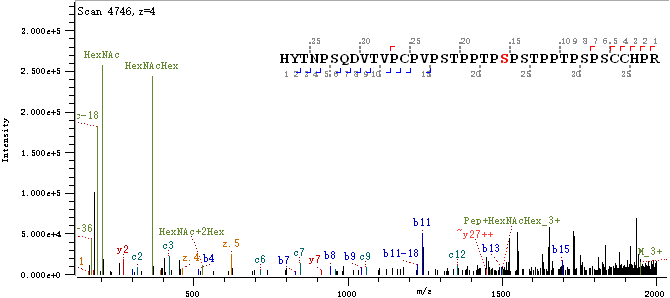


1. **The MS2 spectrum of GalNAc8Gal3 glycopeptide in IgA1 HR.**

GalNAc8Gal3 m/z=1055.4694 z＝5 Retention Time (min)＝18.68

XIC Area =4.65e+8 score=797.87 Error (ppm) =0.90


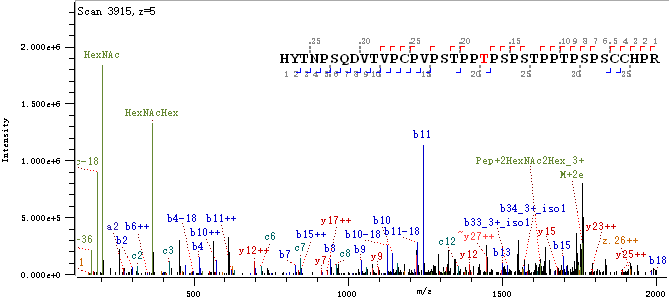


1. **The MS2 spectrum of GalNAc8Gal4 glycopeptide in IgA1 HR.**

GalNAc8Gal4 m/z=1285.9540 z＝5 Retention Time (min)＝18.20

XIC Area =2.4e+8 score=477.96 Error (ppm) =1.50


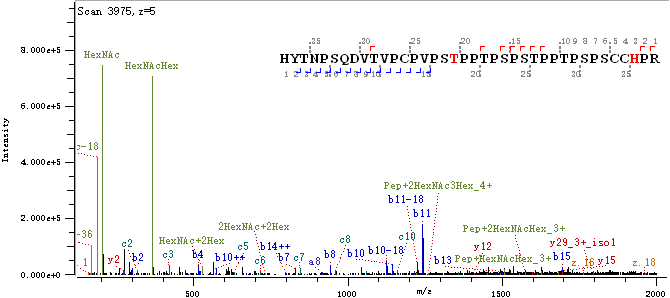


1. **The MS2 spectrum of GalNAc8Gal5 glycopeptide in IgA1 HR.**

GalNAc8Gal5 m/z=1439.6316 z＝5 Retention Time (min)＝16.92

XIC Area =2.84e+7 score=430.08 Error (ppm) =-2.10


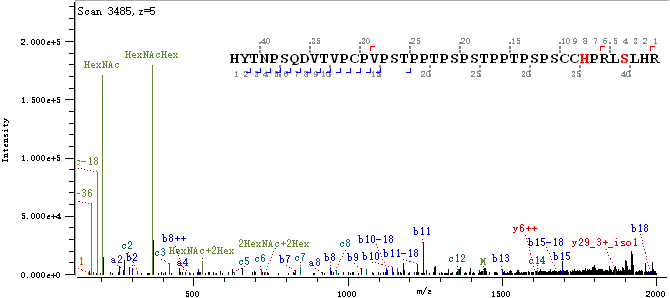


1. **The MS2 spectrum of GalNAc8Gal6 glycopeptide in IgA1 HR.**

GalNAc8Gal6 m/z=1034.9800 z＝4 Retention Time (min)＝21.25

XIC Area =6.11e+7 score=931.48 Error (ppm) =2.02


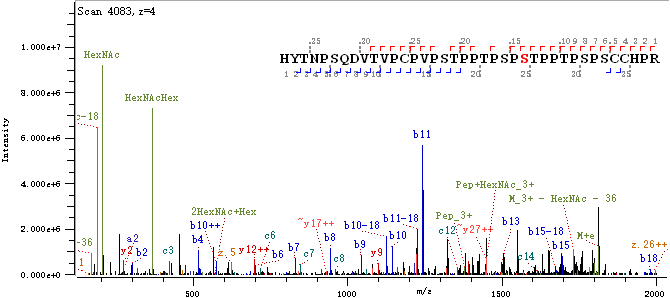


1. **The MS2 spectrum of GalNAc9 glycopeptide in IgA1 HR.**

GalNAc9 m/z=1193.3235 z＝5 Retention Time (min)＝24.11

XIC Area =2.75e+7 score=556.94 Error (ppm) =6.32


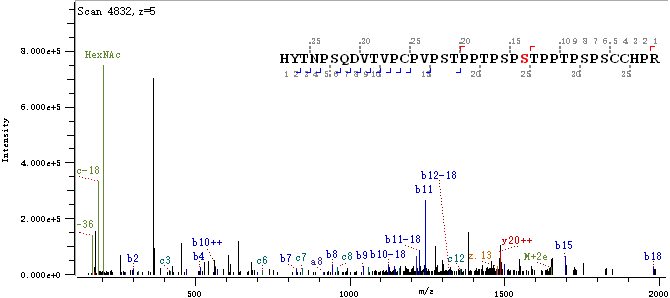


1. **The MS2 spectrum of GalNAc9Gal1 glycopeptide in IgA1 HR.**

GalNAc9Gal1 m/z=1021.9482 z＝6 Retention Time (min)＝18.35

XIC Area =8.21e+7 score=304.72 Error (ppm) =-0.67


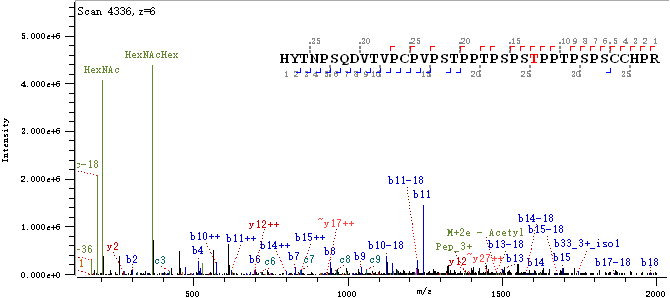


1. **The MS2 spectrum of GalNAc9Gal2 glycopeptide in IgA1 HR.**

GalNAc9Gal2 m/z=1258.5427 z＝5 Retention Time (min)＝18.10

XIC Area =7.75e+7 score=554.15 Error (ppm) =-4.05


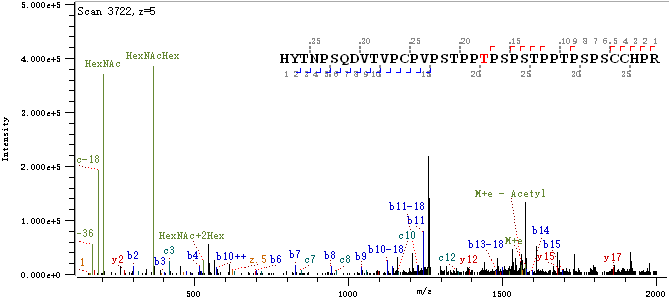


1. **The MS2 spectrum of GalNAc9Gal3 glycopeptide in IgA1 HR.**

GalNAc9Gal3 m/z=1290.9574 z＝5 Retention Time (min)＝17.72

XIC Area =7.64e+7 score=554.17 Error (ppm) = -0.76


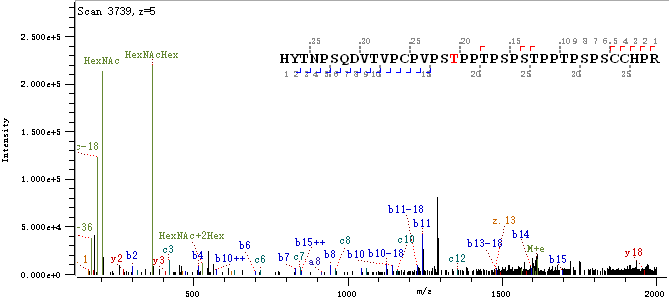


1. **The MS2 spectrum of GalNAc9Gal4 glycopeptide in IgA1 HR.**

GalNAc9Gal4 m/z=1447.8307 z＝5 Retention Time (min)＝21.90

XIC Area =4.81e+6 score=455.64 Error (ppm) =-6.38


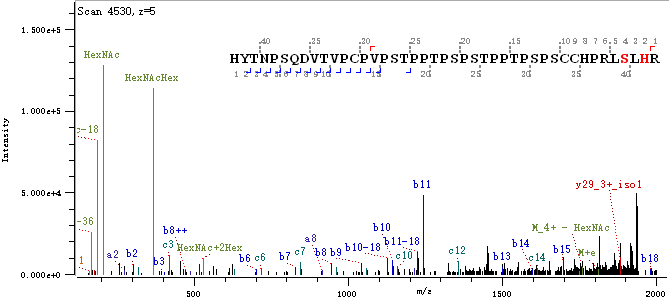


1. **The MS2 spectrum of GalNAc9Ga5 glycopeptide in IgA1 HR.**

GalNAc9Gal5 m/z=1129.9889 z＝6 Retention Time (min)＝21.58

XIC Area =5.38e+7 score=488.84 Error (ppm) =4.26


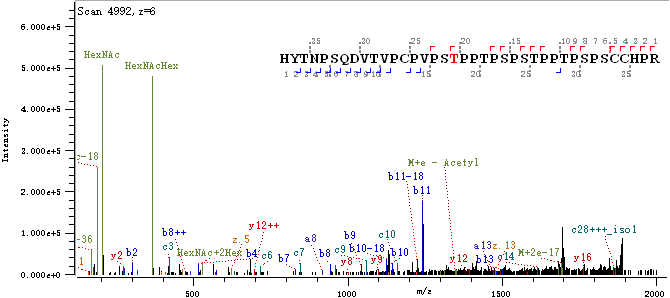

Supplement: Supplementary file 2 [file DataSheet1.DOC]
