# Supplementary material for: Diagnostic Potential of Plasma IgA1 O-Glycans in Discriminating IgA Nephropathy From Other Glomerular Diseases and Healthy Participants
Source: Front Mol Biosci. 2022 Apr 4;9:871615. doi: 10.3389/fmolb.2022.871615 (PMC9014244; doi:10.3389/fmolb.2022.871615)
Supplement: Supplementary file 4 [file DataSheet2.DOCX]

**Diagnostic Potential of Plasma IgA** **O-glycans in Discriminating IgA Nephropathy from Other Glomerular Diseases and Healthy Subjects**

Shuyu Zhang1#; Haidan Sun2; Zejian Zhang3; Menglin Li4; Zhengguang Guo2; Guangyan Cai5*; Wei Sun2*; Mingxi Li1*

1. Department of Nephrology, State Key Laboratory of Complex Severe and Rare Diseases, Peking Union Medical College Hospital, Chinese Academy of Medical Science and Peking Union Medical College, Beijing, China.
2. Core Facility of Instruments, Institute of Basic Medical Sciences, Chinese Academy of Medical Sciences, School of Basic Medicine, Peking Union Medical College, Dong Dan San Tiao, Beijing, China.
3. Medical Research Center, State Key Laboratory of Complex Severe and Rare Diseases, Peking Union Medical College Hospital, Chinese Academy of Medical Science and Peking Union Medical College, Beijing, China.
4. State Key Laboratory of Bioactive Substances and Functions of Natural Medicines, Institute of Materia Medica, Chinese Academy of Medical Sciences, Peking Union Medical College, Beijing, China.
5. Department of Nephrology, The First Medical Centre, Chinese PLA General Hospital, Medical School of Chinese PLA, Beijing, China.

**Corresponding author**

**Mingxi Li1***

Department of Nephrology, State Key Laboratory of Complex Severe and Rare Diseases, Peking Union Medical College Hospital, Chinese Academy of Medical Science and Peking Union Medical College, Beijing, China.

No.1 Shuaifuyuan Street, Beijing, 100730, China

Tel: 8610-65295058

mail: [mingxili@hotmail.com](mailto:mingxili@hotmail.com)

**Wei Sun2***

Core Facility of Instruments, Institute of Basic Medical Sciences, Chinese Academy of Medical Sciences, School of Basic Medicine, Peking Union Medical College, Beijing, China.

Dong Dan San Tiao, Beijing, 100005, China.

mail: sunwei1018@hotmail.com

**Guangyan Cai5***

Department of Nephrology, The First Medical Centre, Chinese PLA General Hospital, Medical School of Chinese PLA, Beijing, China.

E-mail: caiguangyan@sina.com

**Supplementary Materials**

**Chemicals and instruments**

The acetonitrile (ACN), formic acid, trifluoroacetic acid (TFA), ammonium bicarbonate, iodoacetamide (IAA), and dithiothreitol (DTT) were purchased from Sigma (St. Louis, MO, USA). The sequencing-grade trypsin was obtained from Promega (Madison, WI, USA). The peptide-N-glycosidase F (PNGase F) and neuraminidase were purchased from New England Biolabscompany (N2876, Sigma-Aldrich, USA). The ultrafiltration cartridges (30kDa centrifugal filter units) were purchased from PALL Life Sciences (New York, USA). IgA-capture beads (CaptureSelect IgA Affinity Matrix) were obtained from Thermo (Bleiswijk, Netherland). The pipet tips were purchased from Axygen (New York, USA). Venusil HILIC sorbent was provided by Agela Technologies (Tianjin, China). Sep-Pack C18 cartridge was obtained from Waters Corporation (Milford, MA, USA). KM55 kit was purchased from Immuno-Biological Laboratories (#27600, Fujioka, Japan). All other chemicals and reagents were purchased from Sigma-Aldrich or Thermo Fisher Scientific. An Orbitrap Fusion Lumos Tribrid mass spectrometer and an EASY-nLC 1000 HPLC system were purchased from Thermo (Thermo Fisher Scientific, MA, USA).

**Formulae**

The following formulae were used to calculate the glycosylation traits for O-glycosylation of IgA1.

**Number of N in the O- glycosylation of IgA1 HR**

=1*(N1H1+N1)+2*(N2H2+N2H1+N2)+3*(N3H3+N3H2+N3H1+N3)+4*(N4H4+N4H3+N4H2+N4H1+N4)+5*(N5H5+N5H4+N5H3+N5H2+N5H1+N5)+6*(N6H6+N6H5+N6H4+N6H3+N6H2+N6H1+N6)+7*(N7H7+N7H6+N7H5+N7H4+N7H3+N7H2+N7H1+N7)+8*(N8H8+N8H7+N8H6+N8H5+N8H4+N8H3+N8H2+N8H1+N8)+9*(N9H9+N9H8+N9H7+N9H6+N9H5+N9H4+N9H3+N9H2+N9H1+N9)

**Number of H in the O- glycosylation of IgA1 HR**

=1*(N1H1+N2H1+N3H1+N4H1+N5H1+N6H1+N7H1+N8H1+N9H1)+2*(N2H2+N3H2+N4H2+N5H2+N6H2+N7H2+N8H2+N9H2)+3*(N3H3+N4H3+N5H3+N6H3+N7H3+N8H3+N9H3)+4*(N4H4+N5H4+N6H4+N7H4+N8H4+N9H4)+5*(N5H5+N6H5+N7H5+N8H5+N9H5)+6*(N6H6+N7H6+N8H6+N9H6)+7*(N7H7+N8H7+N9H7)+8*(N8H8+N9H8)+9*N9H9

Abbreviations: N=N-acetylgalactosamine(GalNAc), H=glactose(Gal).

**Reference**

[1] Bondt A, Nicolardi S, Jansen BC, Stavenhagen K, Blank D, Kammeijer GS, Kozak RP, Fernandes DL, Hensbergen PJ, Hazes JM, van der Burgt YE, Dolhain RJ, Wuhrer M. [Longitudinal monitoring of immunoglobulin A glycosylation during pregnancy by simultaneous MALDI-FTICR-MS analysis of N- and O-glycopeptides.](https://pubmed.ncbi.nlm.nih.gov/27302155/) *Sci Rep.* 2016,6:27955.

[2] Albert Bondt, Simone Nicolardi, Bas C. Jansen, T. Martijn Kuijper, Johanna M. W. Hazes,Yuri E. M. van der Burgt, Manfred Wuhrer and Radboud J. E. M. Dolhain. IgA N- and O-glycosylation profiling reveals no association with the pregnancy-related improvement in rheumatoid arthritis.*Arthritis Research & Therap.* 2017. 19(1):160.
